# Exposure to Sound Vibrations Lead to Transcriptomic, Proteomic and Hormonal Changes in Arabidopsis

**DOI:** 10.1038/srep33370

**Published:** 2016-09-26

**Authors:** Ritesh Ghosh, Ratnesh Chandra Mishra, Bosung Choi, Young Sang Kwon, Dong Won Bae, Soo-Chul Park, Mi-Jeong Jeong, Hanhong Bae

**Affiliations:** 1 Department of Biotechnology, Yeungnam University, Gyeongsan 38541, Republic of Korea; 2 Environmental Biology and Chemistry Center, Korea Institute of Toxicology, Jinju 52834, Republic of Korea; 3 Central Instrument Facility, Gyeongsang National University, Jinju 52828, Republic of Korea; 4 National Institute of Agricultural Sciences, Rural Development Administration, Wanju 55365, Republic of Korea

## Abstract

Sound vibration (SV) is considered as an external mechanical force that modulates plant growth and development like other mechanical stimuli (e.g., wind, rain, touch and vibration). A number of previous and recent studies reported developmental responses in plants tailored against SV of varied frequencies. This strongly suggests the existence of sophisticated molecular mechanisms for SV perception and signal transduction. Despite this there exists a huge gap in our understanding regarding the SV-mediated molecular alterations, which is a prerequisite to gain insight into SV-mediated plant development. Herein, we investigated the global gene expression changes in *Arabidopsis thaliana* upon treatment with five different single frequencies of SV at constant amplitude for 1 h. As a next step, we also studied the SV-mediated proteomic changes in Arabidopsis. Data suggested that like other stimuli, SV also activated signature cellular events, for example, scavenging of reactive oxygen species (ROS), alteration of primary metabolism, and hormonal signaling. Phytohormonal analysis indicated that SV-mediated responses were, in part, modulated by specific alterations in phytohormone levels; especially salicylic acid (SA). Notably, several touch regulated genes were also up-regulated by SV treatment suggesting a possible molecular crosstalk among the two mechanical stimuli, sound and touch. Overall, these results provide a molecular basis to SV triggered global transcriptomic, proteomic and hormonal changes in plant.

Due to their inability to move, plants are under continuous pressure to deal with copious environmental cues for their successful survival. Therefore, plants have developed numerous sophisticated mechanisms to utilize those cues for modulation of their growth and development in the course of evolution. As a consequence of the incessant evolutionary pressure, plants have developed sensitivity even towards the physical and mechanical factors (e.g., wind, rain, touch, and vibration), besides the well-known biotic and abiotic stimuli (e.g., light, temperature and pathogen attack). The collective term given for plant growth and development in response to mechanical perturbation is thigmomorphogenesis^1^. Thigmomorphogenetic responses, which include stunted growth, increase resistance to other stress, alteration in flowering time, senescence, and chlorophyll contents^1^, are associated with differential expression of specific genes and proteins. Among these, touch genes (*TCH*) are well-known for its touch-mediated rapid up-regulation^2^.

All across the biosphere, every niche inhabited by plants is full of vibrations produced by sound of biological and/or physical origin. Thus, it is undeniable that plants have evolved sensitivity also towards sound vibration (SV) of various ecologically relevant frequencies. A direct evidence to this is the elicitation of defense response in Arabidopsis that leads to accumulation of defense molecules upon treatment with vibrations caused by churning sound of caterpillar^3^. Another classical example is the well-known phenomenon of ‘Buzz Pollination’, where pollens from anthers are released only upon vibration at a particular frequency produced by bee buzz^4^. Sound is a vibrational mechanical force and its effect on plants is an emerging area of research for past few years. In preliminary studies, beneficial effect of music on plants was claimed by many researchers though it has been a subject of debate for decades^5^. Researchers argued that music is a complex mixture of various frequencies, amplitudes and tones, and has neither evolutionary nor ecological significance; however, certain natural music (referred as green music), like bird’s singing, cricket’s stridulating and bee’s buzz etc., may be important^6^. Green music itself has been shown to increase oxygen uptake and polyamine content in chinese cabbage^7^. To avoid the controversy, in subsequent work, many researchers preferred single frequency acoustic vibration. It has already been noticed that ultra and infrasound [above and below the audible range of 20–20000 Hertz (Hz), respectively] can interact with biological tissues^8^. Previously, many researchers have shown beneficial effect of ultrasound on callus regeneration, suspension culture, and plant transformation^9^. By virtue of its growth enhancing effect, SV has been widely implicated in agriculture for facilitating crop growth and yield. Employing plant acoustic frequency technology (PAFT), increased photosynthetic characteristics, growth and disease resistance were achieved in various vegetables^8^. Sound in the range of audible frequencies was also reported to change developmental and physiological processes of plants (e.g., growth, seed germination, cell cycle progression, and plasma membrane architecture)^10^.

Whereas SV application in agriculture/biotechnology has been an area of much interest, studies suggesting the molecular and physiological effect of SV at cellular level are still at infancy. SV has been reported to increase protective enzyme activities, cellular sugar, soluble protein, ATP content, and hormones in many different plant species^8^,^9^,^10^. There are few reports suggesting SV-mediated up-regulation of certain specific genes, like *CAT* encoding catalase, *PAL* encoding phenylalanine ammonia-lyase in hazel, *TCHs* encoding calmodulin-related proteins and xyloglucan endotransglycosylase/hydrolase in Arabidopsis, *RBCS* encoding ribulose-1,5-bisphosphate carboxylase/oxygenase (RuBisCO) small subunit, and *ALD* encoding aldolase in rice^11^,^12^,^13^. Previous study has shown that maize seedlings can indeed generate sound in response to SV treatment of specific frequency^14^. Additionally, bending of roots of maize seedlings towards the sound source was also shown^14^. However, more focused research in the future course is required to explore this new area of plant biology.

In the light of aforementioned studies, it is amply clear that there exist sophisticated mechanisms in plants for SV perception and associated signal transduction. Although the above studies suggest that SV has a big impact on plant physiology, these are only preliminary and insufficient to answer many questions at the cellular level. Thus, there exists a huge gap in our understanding of cellular events triggered upon sound stimulation in plants, which warrants more extensive studies employing the current sophisticated technologies. Herein, we investigated the global transcriptomic and proteomic changes in Arabidopsis upon exposure to SV of five single frequencies with constant amplitude to bridge this gap. Additionally, we deciphered the SV-mediated changes in phytohormone levels. We forwarded a model of SV-mediated transcriptomic, proteomic and hormonal changes in plant, based on the obtained results.

## Results

### Global Gene Expression Profiling after SV Treatments with Five Different Frequencies

**Figure 1:**
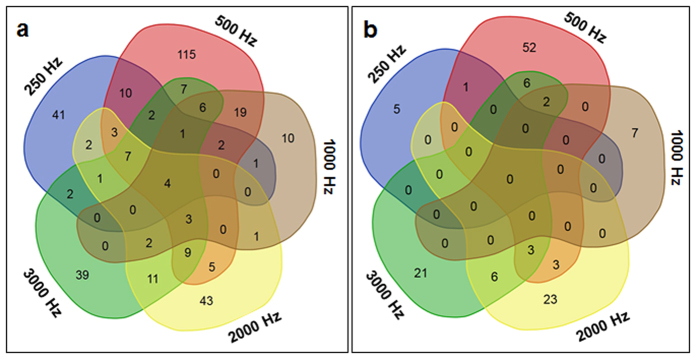
Venn diagram of deferentially expressed genes identified by microarray. (**a**) Venn diagram drawn based on more than 2-fold up-regulated genes (*P* < 0.05). (**b**) Venn diagram drawn based on more than 2-fold down-regulated genes (*P* < 0.05). Five different frequencies (250, 500, 1000, 2000 and 3000 Hertz) were separately applied to Arabidopsis for 1 h with 80 decibels.

**Figure 2:**
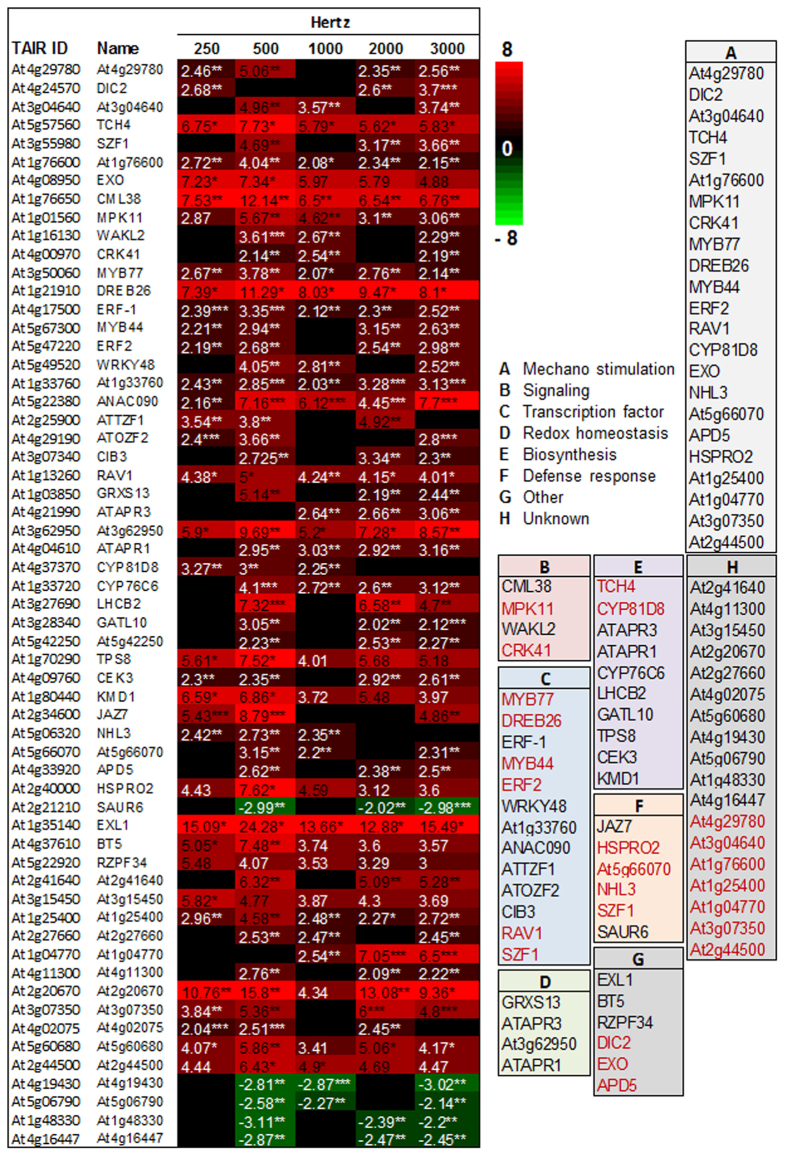
Heatmap of selected genes which showed more than 2-fold differential expression in microarray. The selected genes were common between three treatments of frequencies at least. Color code and numbers represent the fold change (*n* = 3) compared to control. Classification is marked by uppercase letter in side panel. Touch-regulated genes are marked in red color. *P*-value ranges are marked by asterisks: ***P < 0.01, **0.01 < *P* < 0.05, *P < 0.1.

For microarray analysis, samples were harvested right after the SV treatments of 1 h (0 h time point) to check its immediate effect on global transcriptome (Supplementary Fig. S1). Microarray profiling of Arabidopsis plants treated separately with 5 different frequencies (250, 500, 1000, 2000 and 3000 Hz) at constant amplitude (80 dB) suggested differential expression of various genes (Supplementary Table S1). Maximum number of genes was differentially expressed especially by 500 Hz treatment. We selected all the genes which showed more than 2-fold differential expression (*P* < *0.05*) and prepared a Venn diagram (Fig. 1). While four genes (At5g22380, At1g76650, At4g17500, and At1g33760) were found to be up-regulated commonly by five frequencies, there was not even a single gene that was commonly down-regulated at *P* < 0.05. Two genes (At4g19430 and At5g06790) were commonly down-regulated by 500, 1000 and 3000 Hz treatments. Three genes (At4g16447, At1g48330 and At2g21210) were down-regulated by 500, 2000, 3000 Hz. Treatment with 500 Hz shared maximum number of differentially expressed common genes with other Hz treatments. We next performed Gene Ontology (GO) analysis to characterize differentially expressed genes. According to GO analysis most of the differentially expressed genes are nuclear localized (Supplementary Fig. S2a). Genes localized to plastid, cytoplasm and plasma membrane are also large in number. Functionally, many of them have transferase and kinase activities and work as transcription factors (TFs) (Supplementary Fig. S2b). Further, majority of the genes were found to be involved in stress responses and signal transduction pathways (Supplementary Fig. S2c). Next, we chose 59 genes which were differentially expressed in at least three of the given treatments with a fold change >2 to prepare heatmap (Fig. 2; Supplementary Table S2). Heatmap clearly indicated that maximum gene up-regulation occurred by 500 Hz treatment. Based on their attributes, we classified these 59 genes into following groups.

### Group A (Mechanostimulus responsive genes)

Lee *et al*. have shown touch-induced expression of 589 genes (around 2.5% of total genes) in Arabidopsis through microarray analysis^15^. Twenty-three touch regulated genes were also up-regulated by SV treatments, which are marked as mechanostimulus responsive genes in this group (Fig. 2). *TCH4*, which is a well-known marker for touch response^2^, showed high up-regulation especially by 500 Hz treatment. At1g21910 (*DREB26*, encoding an AP2 type TF) showed highest up-regulation by all the treatments. Notably, several genes categorized in this group are involved in other biological process and/or have different functional role. Thus, those genes are mentioned in the other groups as well.

### Group B (Signaling related genes)

Among the up-regulated genes, four were noted to function in cell signaling. These genes are At1g76650 (*CML38*), At1g01560 (*MPK11*), At1g16130 (*WAKL2*), and At4g00970 (*CRK41*). *MPK11* and *CRK41* are also marked as mechanostimulus responsive genes in the group A (Fig. 2). The one that showed the highest up-regulation is *CML38*, encoding Ca^2+^ binding calmodulin-like protein. The other three genes (*MPK11, WAKL2* and *CRK41*) are kinase family members. These were highly up-regulated by 500 and 1000 Hz treatments.

### Group C (Transcription factors)

Total thirteen genes that comprise a variety of TFs constitute this group. Out of these 13 genes, 6 TFs are marked as mechanostimulus responsive in the group A: namely: *DREB26*, At3g50060 (*MYB77*), At5g67300 (*MYB44*), At5g47220 (*ERF2*), At3g55980 (*SZF1*), and At1g13260 (*RAV1*). Two of the TFs that showed very high up-regulation at all treatments are *DREB26* and At5g22380 (*ANAC090*). This group also includes two MYB TFs *MYB77* (encoding an R2R3 type TF) and *MYB44*, which were significantly up-regulated by SV treatments. Also, three ethylene response factor (ERF) family members [At4g17500 (*ERF1*)*, ERF2* and At1g33760], and three C3H family members [*SZF1,* At2g25900 (*ATTZF1*) and At4g29190 (*ATOZF2*)] were up-regulated by SV treatments. Besides, At5g49520 (*WRKY48*) and At3g07340 (*CIB3,* encoding bHLH) family members were also up-regulated. *RAV1*, which encodes a TF of APETALA2/ERF (AP2/ERF) family^16^, showed up-regulation in our study.

### Group D (Redox homeostasis)

Four of the up-regulated genes were noted to function in cell redox homeostasis. These are At1g03850 (*GRXS13*), At4g04610 (*ATAPR1*), At4g21990 (*ATAPR3*) and At3g62950. At3g62950, encoding a member of thioredoxin (TRX) superfamily, showed the highest up-regulation by all the treatments. *ATAPR1* and *ATAPR3* that encode protein disulfide isomerase-like (PDIL) proteins are also members of TRX superfamily. *GRXS13* that encodes a member of glutaredoxin (GRX) family showed high up-regulation specifically by 500 Hz.

### Group E (Biosynthesis)

Many differentially expressed genes fall into this group. Interestingly, 2 mechanostimulus responsive genes [*TCH4*, At4g37370 (*CYP81D8*); in group A] and 2 redox homeostasis related genes (*ATAPR1* and *ATAPR3*; in group D) are involved in cellular biosynthetic process and thus are also categorized in this group (Fig. 2). Photosynthesis related gene At3g27690 (*LHCB2*) was up-regulated by 500, 2000 and 3000 Hz. Two genes, At3g28340 (*GATL10*) and At1g70290 (*TPS8*) that are related to carbohydrate metabolism were also up-regulated. *TPS8* encodes a type II trehalose-6-phosphatase synthase, which is involved in biosynthesis of trehalose, an osmoprotectant^17^. TCH4 involves in cell wall biogenesis through xyloglucan metabolism^1^. Two sulfur metabolism related genes (*ATAPR1* and *ATAPR3*)^18^, showed up-regulation specifically at higher frequencies (1000, 2000 and 3000 Hz). APS reductase (APR) also involves in cysteine and glucosinolate biosynthesis network^19^. *CYP81D8* and At1g33720 (*CYP76C6*) which encode important cytochrome P450 family proteins were up-regulated by SV treatments. According to Kyoto Encyclopedia of Genes and Genomes (KEGG) orthology, CYP81D8 is involved in stilbenoid, diarylheptanoid and gingerol biosynthesis. Cytochrome P450 family members are very important for various metabolic process, like hormone, alkaloids, terpeniods and glucosinolates^20^. At4g09760 (*CEK3*), which is a member of choline/ethanolamine kinase family, is involved in phospholipid biosynthesis^21^. At1g80440 (*KMD1*), which encodes a Kelch repeat F-box (KFB) protein and involved in phenylpropanoid pathway^22^, was upregulated by all Hz, especially 500 Hz.

### Group F (Defense related genes)

Many defense related genes were also differentially expressed after SV treatments. Among them, 4 genes [At2g40000 (*HSPRO2*), At5g66070, At5g06320 (*NHL3*) and *SZF1*] being mechanostimulus responsive are also categorized in group A (Fig. 2). Two of the defense related genes showed the highest up-regulation by 500 Hz: (1) At2g34600 (*JAZ7*), encoding a transcription repressor protein that regulates jasmonic acid (JA)-mediated defense response^23^, and (2) *HSPRO2*, encoding salicylic acid (SA)-responsive positive regulator of basal resistance^24^. At5g66070 that encodes a chitin responsive ubiquitin-ligase-like enzyme, showed maximum up-regulation specifically by 500 Hz^25^. Chitin is known as pathogen-associated molecular patterns (PAMP) which sensitize the plant immune system^25^. At2g21210 (*SAUR6*), which is also chitin- responsive according to GO, was down-regulated by SV treatments. The touch-responsive TF SZF1 is also chitin- responsive and showed SV-mediated up-regulation. *NHL3*, a positive regulator of plant defense, was up-regulated by SV treatment^26^.

### Group G (Others)

The genes in this group are involved in diverse cellular functions. At1g35410 (*EXL1*), which is involved in plant growth during carbon starvation^27^, was highly up-regulated by all treatments. A ‘BTB AND TAZ’ domain protein (At4g37610, *BT5*) was highly up-regulated by 500 Hz. At5g22920 (*RZF34*), involved in stomatal opening process^28^, was induced by SV treatments. At4g24570 (*DIC2*) belongs to mitochondrial carrier protein family and involved in membrane transport^29^. At4g08950 (*EXO*), which is involved in cell expansion of Arabidopsis leaves, was upregulated by all Hz^30^. At4g33920 (*APD5*), which encodes a protein phosphatase 2C family protein, was up-regulated significantly by all treatments except 250 and 1000 Hz. *DIC2*, *APD5,* and *EXO*, are mechanostimulus responsive and thus categorized in group A as well (Fig. 2).

### Group H (Unknown)

Genes, which are not defined by any GO biological process, are placed in this group. Among them At2g20670 was highly induced by all SV treatments whereas At4g19430, At5g06790, At1g48330 and At4g16447 were down-regulated. Seven mechanostimulus responsive genes are also categorized in this group (Fig. 2).

Notably, many of the aforementioned genes discussed under different groups are also functioned in hormone biosynthesis and/or signaling pathways. A brassinosteriod (BR) responsive gene *EXO*, which encodes for an extracellular protein involved in cell expansion^30^, was highly up-regulated by all treatments. The TFs *ERF1* and *ERF2* are important players in ethylene signaling pathway^31^. *DREB26, MYB77* and *MYB44* are also responsive to SA according to GO. Additionally, MYB77 is also involved in auxin signaling pathway by directly interacting with auxin response factors (ARFs)^32^. Like *EXO*, *RAV1* is another BR stimulated gene. Furthermore, RAV1 is known as a negative regulator of abscisic acid (ABA) signaling during seed germination and early seedling stage^16^.

### Validation by quantitative real-time PCR (qRT-PCR)

**Figure 3:**
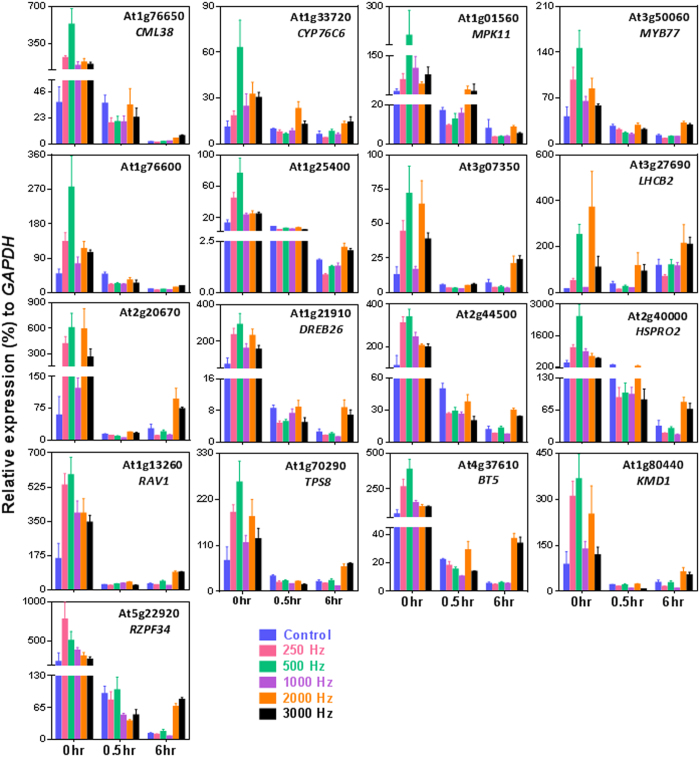
Quantitative real-time PCR confirmation of selected genes. Error bar indicates the standard error of means from four biological replications. Statistical analysis (Duncan’s multiple range test, DMRT) are mentioned in Supplementary Table S3.

To validate microarray results, qRT-PCR was done immediately after SV treatments (0 h). Additionally, two more time points (0.5 and 6 h) were chosen for qRT-PCR analysis in order to reveal the trend in the expression pattern of genes, which were differentially regulated at 0 h time point. Expression of total seventeen genes was validated (Fig. 3; Supplementary Table S3). These seventeen genes are involved in diverse function. For a quick reference, the fold change based on microarray results and functions of these seventeen genes are mentioned in Table 1. Among these, nine genes (*MYB77*, *DREB26*, *HSPRO2*, *RAV1*, *MPK11*, At3g07350, At2g44500, At1g76600 and At1g25400) are touch regulated^15^. By and large, the qRT-PCR profiling was noted to be in good correlation with microarray results. In qRT-PCR analysis, maximum up-regulation of all the selected genes was noted by 500 Hz at 0 h. Gene that showed the highest up-regulation at this condition was *HSPRO2*. Moving from 0 to 0.5 h, a decline was noted in the expression levels of all the genes; except for *CYP76C6*, *MPK11* and *LHCB2,* which remained significantly up-regulated by 2000 or 3000 Hz at 0.5 h. The expression of the rest of the genes declined, in large, to control levels. At 6 h, significant differential expression was noted for fourteen genes only by 2000 and 3000 Hz treatments. In summary, (1) genes were maximally induced at 0 h by all the treatments with most of the genes showing the highest up-regulation by 500 Hz, (2) at 0.5 h, gene expression declined to the control levels, and (3) at 6 h, most of the genes again showed up-regulation by 2000 and 3000 Hz treatments.

### Global Proteomic Profiling after SV Treatments with Five Different Frequencies

**Figure 4:**
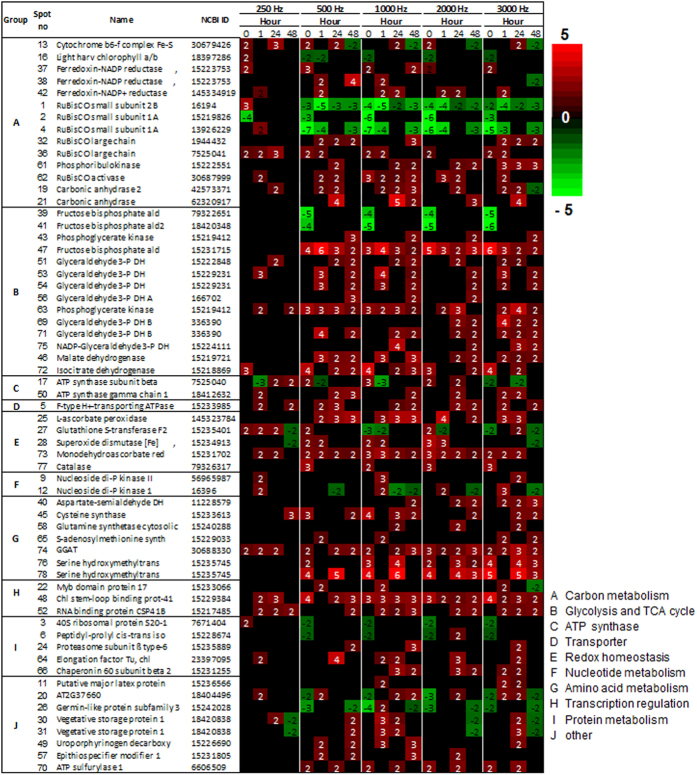
Heatmap of selected proteins identified by MALDI-TOF/MS. Color code and numbers represent the significant fold change (*n* = 3, *P* < 0.05) compared to control. Classification is marked by uppercase letter. Proteins were classified based on Mapman annotation of the plant proteome database (PPDB). Functional annotations of proteins are mentioned in Supplementary Table S7.

To analyze the instantaneous effect of SV exposure on protein expression, two early time points were chosen for proteomic analysis: 0 and 1 h after the SV treatments. Also, two later time points – 24 and 48 h – were chosen to reveal the delayed effects of SV treatments on protein expression (Supplementary Fig. S1). For proteomic analysis, two-dimensional gel electrophoresis (2-DGE) followed by protein identification by matrix-assisted laser desorption/ionization mass spectrometry (MALDI-TOF/MS) and MALDI-TOF/TOF MS were performed (Supplementary Fig. S3; Tables S4 and S5). Differentially expressed proteins between control and treatment were classified by using online GO platform. Among the differentially expressed proteins, most proteins turned out to be involved in carbohydrate metabolism and photosynthesis (Supplementary Fig. S4a). The third largest group is constituted by stress and defense related proteins. As suggested by GO analysis, many of the differentially expressed proteins are either plastidic or apoplastic in localization (Supplementary Fig. S4b). Further, the differential expression of proteins having antioxidant function was most significant (Supplementary Fig. S4c). Fold changes and functional annotations of differentially expressed proteins are available in supplementary Tables S6 and S7, respectively. For the ease of understanding, we categorize the differentially expressed proteins on the basis of their function into following categories (Fig. 4).

### Photosynthesis

RuBisCO is the most important enzyme involved in the first major step of carbon fixation. It consists of two types of protein subunits, the large chain and the small chain. We noted up-regulation of two large chain members of RuBisCO (RBCL) in our analysis. However, three small subunits of RuBisCO (RBCS) were significantly down-regulated by all the treatments, except 250 Hz. Phosphoribulokinase (PRK), an enzyme involved in Calvin cycle, was up-regulated at 24 and 48 h with the highest up-regulation by 3000 Hz. A light harvesting complex protein (LHCB, spot 16) showed up-regulation immediately after 250 Hz treatment at 0 h whereas the same protein down-regulated at 0 h by other Hz. Glutamate-glyoxylate aminotransferase 1 (GGAT), which is an important enzyme in photosynthetic carbon fixation cycle, photorespiration and metabolism of major amino acids, was up-regulated by all treatments, largely, at every time points. The cytochrome b6-f complex was upregulated at 0 h by 250, 500 and 2000 Hz. Also, it was up-regulated at 24 h by most of the treatments. However, it was down-regulated at 48 h by all the treatments, except 250 Hz. Carbonic anhydrase (CA), which is an important enzyme for decarboxylation/carboxylation reactions in carbon- assimilation and other physiological process^33^, showed increased expression by all treatments.

### Respiration

Around 24% of the differentially expressed proteins constitute this group. A fructose-bisphosphate aldolase (FBPA, spot 47), which is an important enzyme of glycolytic/gluconeogenic pathway in plants, was significantly up-regulated at each time point by all treatments except 250 Hz. However, two FBPA (spots 39, 41) were down-regulated at 0 h by all treatments except 250 Hz. Several glyceraldehyde-3-phosphate dehydrogenase (GAPDH) proteins were also significantly up-regulated. Phosphoglycerate kinase (PGK), involved in glycolysis pathway, was up-regulated especially by 500 and 1000 Hz. The two important enzymes of TCA cycle, (1) malate dehydrogenase (MDH) and (2) isocitrate dehydrogenase (IDH), were also up-regulated.

### Amino acid biosynthesis

Aspartate-semialdehyde dehydrogenase (ASADH), the enzyme linked to homoserine and threonine biosynthesis pathway was up-regulated by 1000, 2000 and 3000 Hz. Similarly, cysteine synthase (CS) and S-adenosylmethionine synthase (SAMS), enzymes involved in biosynthesis of cysteine and methionine, respectively, were strongly up-regulated by SV treatment. Enzymes glutamine synthetase (GS) and glutamate glyoxylate aminotransferase (GGAT) were also strongly up-regulated. These enzymes are interlinked in glutamate, glutamine and glycine biosynthesis during photorespiratory nitrogen cycle. Another important enzyme, serine hydroxymethyltransferase (SHMT), which is critical for serine and glycine metabolism, also showed significant up-regulation by all treatments, except 250 Hz.

### Energy metabolism

Notably, two ATP synthase proteins were also differential expressed after SV treatment. Among them, γ chain protein was significantly up-regulated, whereas β chain showed mixed expression. While γ chain protein was highly up-regulated by 500 and 1000 Hz at 48 h, β chain showed up-regulation by the same Hz at 0 h. By 1000 Hz treatment, moving from 0 h to 1 h, β chain showed maximum down-regulation.

### Transporter

There are two types of ATPase transporters. V-type is located in vacuole, while F-type is located in chloroplast and mitochondria^34^. While F-type showed more consistent up-regulation by all treatments, V-type was up-regulated only by 1000 Hz at 48 h (Supplementary Table S6).

### ROS scavenger

Maintaining redox homeostasis is of utmost importance for cellular survival both under normal conditions and different environmental stresses. In our proteomic data, we noted differential expression of several ROS scavenging and detoxifying enzymes: ascorbate peroxidase (APX), superoxide dismutase (SOD), monodehydroascorbate reductase (MDAR), catalase (CAT) and glutathione S-transferase (GST). Among these, CAT and SOD were up-regulated at early time points (0 and 1 h). APX also showed significant up-regulation at many time points. MDAR was up-regulated by all treatments, in large, at each time points. MDAR showed maximum up-regulation at 0 h by 2000 Hz treatment.

### Miscellaneous

Nucleoside diphosphate kinase (NDK) is an important enzyme playing role in nucleotide metabolism. We noted increased levels of this enzyme immediately after all treatments at 1 h, except 500 Hz. However, it showed down-regulation at later time points (24 and 48 h). Chloroplast stem-loop binding protein-41 (CSP41, endoribonuclease), critical for regulation of chloroplastic transcription and translation^35^, were up-regulated by all treatments. Another RNA binding protein (CSP41B) also showed elevated expression with maximum level at 48 h after 1000 Hz treatment. Differential expression of a JA-regulated vegetative storage protein 1(VSP1), was also noted^36^. VSP1 was maximally expressed by 1000 Hz. ATP sulfurylase (ATPS), the first enzyme of the sulfate assimilation pathway^18^, was also up-regulated by all treatments. Uroporphyrinogen decarboxylase (UROD), an important enzyme linked to chlorophyll synthesis pathway by virtue of its function in heme biosynthesis^37^, was strongly up-regulated by SV treatments. Several enzymes related to protein metabolism, folding and degradation were also differentially expressed after SV treatments.

### Phytohormonal Profiling after SV Treatments with 500 Hz

From the transcriptomics and proteomics results, it accrued that 500 Hz with 80 dB has maximum impact on cellular processes in Arabidopsis. Moreover, many phytohormone biosynthetic and/or signaling related genes showed significant differential expression by 500 Hz. We thus treated plants with 500 Hz frequency and 80 dB amplitude for phytohormonal analysis. Alteration in hormonal levels is modulated by several synergistic and antagonistic upstream events and thus hormonal interplay comes later in a signaling cascade. To evaluate persistent changes in hormonal levels caused by SV exposure, later time points post-500 Hz treatment – 6, 24 and 48 h – were chosen. Five different phytohormones [ABA, gibberellic acid 3 (GA_3_), indole acetic acid (IAA), JA and SA] were analyzed (Supplementary Fig. S5). We noted significant changes in the levels of all the phytohormones, except ABA. Notably, the levels of the two phytohormones, GA_3_ and auxin (IAA), were significantly increased at 24 h after SV treatment. The level of JA, however, was increased only after 48 h of SV treatment. SA levels were high at all the time points with maximum magnitude after 48 h of treatment. Noticeably, only IAA and SA showed changes in their levels at 6 h after treatment.

## Discussion

Beneficial effects of SV on plant physiology leading to enhanced growth, development and disease resistance are well established by many previous reports^8^,^10^. However, there is evidently no study addressing the detailed molecular events triggered by SV to substantiate these observations. Being prompted by this lacuna in our knowledge, we aimed at analyzing the global transcriptomic, proteomic and phytohormonal changes stimulated by SV in Arabidopsis. When an environmental cue incites a plant, several intercellular events occur in a cascading fashion. All these changes collectively aid plants to bring in favorable adjustments. Hierarchically, stimulus/signal perception, signal transduction and molecular/physiological responses are the three broad episodes associated with plant-stimulus interaction. In our study, we strikingly noted differential regulation of many molecular components associated with these events in Arabidopsis exposed to SV. Responses to stimuli are, in large, essentially accompanied with signature events, like calcium transients, ROS outburst/signaling, alteration in gene expression and proteome, and phytohormonal adjustments etc. Interestingly, our microarray results revealed huge change in the expression profile of genes associated with these signature cellular events providing a glimpse of the overall signaling cascade triggered by SV in plants. Many genes including the ones involved in calcium signaling (CML38), redox homeostasis (TRX and GRX), phosphorylation/dephosphorylation (signal transducer kinases; MPK11, WAKL2 and CRK41) and regulation of transcription (TFs; DREB, ANAC, MYB, ERF, C3H, WRKY and RAV) were differentially expressed. Moreover, a large number of genes involved in hormonal signaling were also up-regulated.

In a previous study, a direct connection between calcium transients and SV-mediated enhancement in callus growth was established through pharmacological studies^38^. This highlighted the importance of calcium in SV-mediated responses. Calcium acts as a second messenger which facilitates a signaling cascade. Interestingly, we noted remarkable up-regulation of *CML38*, encoding calmodulin-like protein. CML is a calcium-binding messenger protein that facilitates downstream signaling^39^. In light of the previous studies, our result thus suggests a possible involvement of the second messenger calcium in signaling of SV stimulus. It is argued that being pressure waves, SV influences cells mechanically^5^. Thus, there may be a crosstalk between the signal transduction mechanism of sound and mechanical stimuli. It was suggested that calcium signaling plays an important role in perception of mechanical stimuli, like touch^40^. Furthermore, the important role of calmodulin (CAM) and CML proteins in touch response was highlighted. Additionally, as our transcript analysis suggested up-regulation of several other touch regulated genes (*DIC2*, *TCH4*, *SZF1, MPK11*, *CRK41*, *MYB77*, *DREB26*, *MYB44*, *ERF2*, *RAV1*, *CYP81D8*, *EXO*, *NHL3*, *APD5* and *HSPRO2*), we propose that SV and touch share some common mechanosensitive signaling events.

Perturbation by external stimuli causes elevation of ROS, which participates in cellular signaling^41^. ROS accumulation above optimal range is highly detrimental for cell; thus, to bring this increased level near optimum several ROS scavenging enzymes are accumulated and/or activated as a counter mechanism. In our proteomic study, we noted strong up-regulation of several antioxidant enzymes, like APX, SOD, MDAR and CAT. This is further substantiated by few available studies, where enhancement in activities of several ROS scavenging enzymes were noted in plant calli and seedlings exposed to SV^13^,^42^. It thus appears that ROS signaling is possibly an important player for SV-mediated molecular changes in the plant cell.

Active transport of important molecules across cell membrane is a prerequisite to maintain a healthy physiological state within a cell. In a previous study, SV has been shown to stimulate activity of plasma membrane H^+^-ATPase^43^. H^+^-ATPase has enormous roles in active transport, stress tolerance, and pH regulation^44^. Notably, proton (H^+^) transport coupled with ATP hydrolysis generates a proton motive force, which is essentially required for active transport of other molecules^44^. Complying with the above report, deferential expression of two different types of H^+^-ATPase was noted in our study. Among them F-type showed consistent up-regulation by various Hz. It has also been observed previously that SV (1000 Hz) can increase permeability of K^+^ channel in chrysanthemum callus^45^. Hamilton *et al*.^46^ has reported mechanosensitive Ca^2+^ and K^+^ channels in plants. Considering that some ion channels have mechanosensing property, activation of ion transporters may have a possible role in the SV-mediated cellular responses.

**Figure 5:**
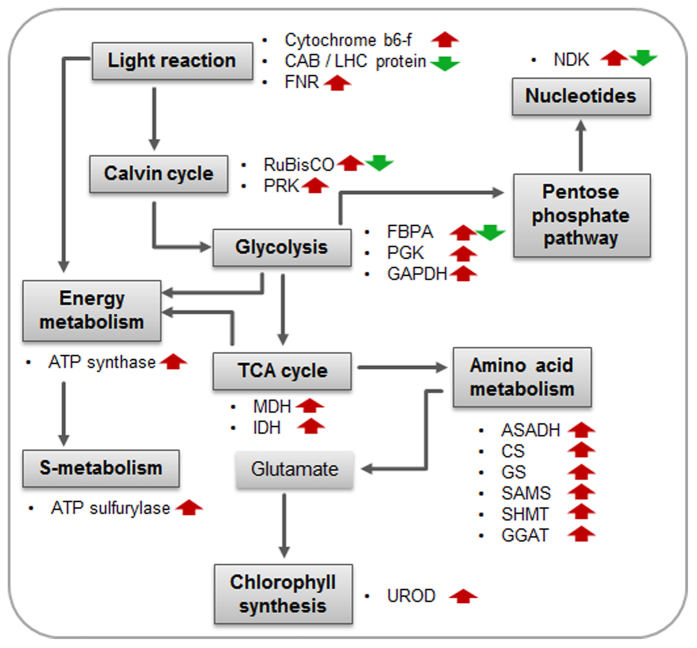
Summary of proteomic changes in primary metabolism. Red and green arrows represent up- and down-regulation, respectively.

Stimulation by an external factor is likely to bring modifications in proteome related to primary metabolism in plants^47^. Reportedly, for rerouting several metabolic pathways and attaining nitrogen/carbon balance, plants alter carbon metabolism^48^. In previous studies, long term audible-SV treatment has been shown to promote plant growth^8^, which indicates that sound brings positive changes in photosynthesis and sugar metabolism. Corroboratively, SV has been noted previously to increase photosynthetic rate and chlorophyll fluorescence in strawberry plant^49^. Accelerated growth also fits well with enhanced carbon metabolism. In line with this, we noted that SV altered expression of several enzymes involved in light reaction, Calvin cycle, glycolysis and TCA cycle, with majority of them being up-regulated (Figs 4 and 5). In a previous study, Jeong *et al*. specifically reported SV-mediated increase in *RBCS* transcript expression in Arabidopsis^12^. RuBisCO is the key regulatory enzyme of C3 carbon fixation and its up-regulation may thus reflect the enhanced photosynthetic state of plants after SV exposure. Contrastingly, RBCL protein was up-regulated in our study. Furthermore, we did not find the up-regulation of RBCS protein. The important thing to notice here is that RBCL is encoded by chloroplast genome whereas RBCS is nuclear encoded; contrasting expression of these subunits suggests that SV can trigger different signaling events between cell organelle. RuBisCO can adopt either carboxylase (photosynthesis) or oxygenase (photorespiration) activity depending on CO_2_ concentration^50^. Increased accumulation of carbonic anhydrase noted in our study thus possibly helps to maintain CO_2_ concentration favorable for enhanced photosynthesis. Besides RuBisCO, we also noticed strong up-regulation of *LHCB2* transcript, which encodes a light harvesting complex playing critical role in providing energy required for photolysis. While LHCB protein mainly showed down-regulation, it is in agreement to the previous findings^51^. Strong up-regulation in Ferredoxin-NADP^+^ reductase (FNR) was marked in our study. FNR is involved in production of NADPH, which is used as reducing power by various metabolic process like Calvin cycle, amino acid biosynthesis, and lipid biosynthesis^52^. Thus an overall shift in the physiological state which is more favorable for efficient photosynthesis is suggested by our results.

Previous research indicates that sound can increase soluble sugar in chrysanthemum root and *Dendranthema morifolium* callus^53^,^54^. In this study, up regulation of *TPS8*, which is involved in trehalose metabolism, also indicates the change of carbohydrate metabolism upon sound. This observation is important to note as soluble sugar itself act as a signaling molecule for activation of hormonal crosstalk and oxidative pentose phosphate (OPP) pathway-mediated ROS scavenging^55^. Alongside, many respiratory genes/proteins were also marked to be up-regulated. Glycolytic-TCA enzymes (e.g., FBPA, PGK, GAPDH, MDH and IDH) that are involved in respiratory process are the prominent ones. Previously, SV was noted to increase the transcripts encoding FBPA in rice^12^. In our study, FBPA showed differential expression among their isoforms (spot 39, 41 and 47 in Fig. 4) suggesting a possibility that different isoforms are involved in varied metabolic processes. Increased expression of the aforementioned glycolytic enzymes suggests efficient breakdown of complex sugar for gaining useful energy to fuel cellular activities. This energy, in large, feeds linked metabolic pathways like amino acid metabolism. Clearly, rerouting metabolic pathway to bring adjustments favorable to an external stimulus requires ample energy. In cellular milieu, the molecular power that can readily be utilized is ATP. In plant cell, ATP is produced by both photophosphorylation in the chloroplast and oxidative phosphorylation in the mitochondrion, which are linked to light reaction, glycolysis and TCA cycle. As discussed above several genes/proteins associated with these processes are up-regulated by SV treatment. Furthermore, we also marked up-regulation in ATP synthase in proteome resulted from SV exposure. In agreement with this idea, SV (500 and 1000 Hz) has already been shown to increase the ATP content in *Actinidia chinensis* callus^56^. Hence increase in ATP synthase, which eventually may results in increased ATP synthesis, could be one of the reasons behind acceleration of metabolic pathways.

Like other external stimuli, SV has also been noted to increase soluble protein contents in plant cells^53^,^54^. Also, to account for the increased levels of several proteins noted in our study, synthesis of amino acids directly from stem pathway or through inter conversion is a prerequisite. In corroboration to this, strong up-regulation of various amino acid biosynthetic enzymes was also noted in our proteome result (Figs 4 and 5). Furthermore, several enzymes related to protein metabolism, folding and degradation were also differentially expressed. Among the up-regulated pool, two important enzymes (ATP sulfurylase and cysteine synthase) showed remarkable up-regulation. ATP sulfurylase is one of the key enzymes for sulfur metabolism^18^, which is responsible for the synthesis of important amino acids, cysteine and methionine. Additionally, up-regulation of the transcripts (*AtAPR1* and *AtAPR3*) involved in sulfur metabolism was also marked in the microarray results. Further, cysteine is an important amino acid for the production of various sulfur containing defense molecules in plants, like sulfur-rich proteins (SRPs), phytoalexins and glucosinolates^19^. Increased synthesis of sulfur containing amino acids may thus be a reason behind the increased disease resistance noted in SV-treated plants in earlier studies^3^.

Plant growth and development in response to an external cue is tightly regulated by phytohormones. In previous studies, SV were noted to bring alteration in hormonal levels^57^,^58^. In the present study, SV exposure resulted into alterations in endogenous phytohormonal levels (Supplementary Fig. S5). Overall, levels of two growth hormones (GA_3_ and IAA) and two defense hormones (JA and SA) were strongly increased at 24 and 48 h, respectively. Interestingly, SA showed accumulation at all three time points. It is important to note here that SA and JA are antagonistic with each other^59^. This possibly is a reason behind opposite accumulation patterns of SA and JA at early time points (6 and 24 h) noted herein. Comparing the levels of all hormones, SA showed high accumulation at all-time points after SV exposure. Enhanced SA level by SV treatment could be the reason for improved disease resistance through ‘priming effect’ in previous study^3^. We discussed in the foregoing text that there exist a possible crosstalk between the touch and sound mechanosensing. Response to mechanical stimuli is itself partly mediated by ethylene and other hormones like auxin, ABA and JA^40^. Notably, touch and other mechanical stimuli showed reduced growth phenotype, which is strongly correlated with mechano-stimulated up-regulation of the negative growth regulator ABA^40^. However, the studies available hitherto suggest growth enhancement in plants upon exposure to SV^8^. Corroboratively, we could not find any changes in ABA level after SV treatment. In addition up-regulation of *RAV1* transcript, a negative regulator of ABA signaling^16^, also indicates that SV-mediated response is probably ABA-independent. The important points emerging here are: (1) SV exposure appears to have less impact on ABA biosynthesis compared to other hormones, (2) besides the possible crosstalk between the signaling of touch and sound, plant perceives SV as an ecologically distinct stimulus and comes up with responses tailored accordingly. Thus, in the future course, detailed hormonal studies are required to establish strong correlation between SV and hormonal modulation.

**Figure 6:**
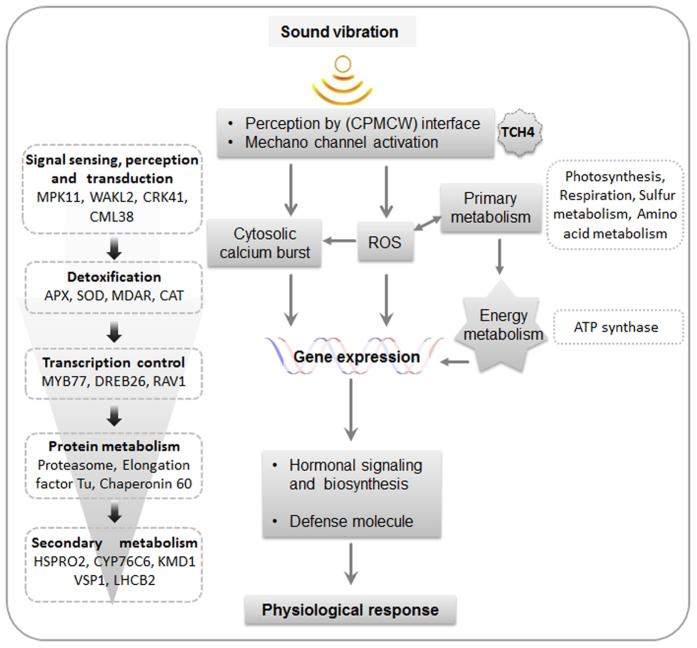
Hypothetical model of sound perception and signal transduction in plant cell. Combining previous reports and our analysis we can predict sound perception mechanism by cytoskeleton-plasma membrane-cell wall (CPMCW) interface. Dotted boxes indicate representative transcripts or proteins identified by our experiment. Arrow marks indicate activation.

Based on the results obtained in our study, we conclude with a model depicting the effect of SV on plant cell (Fig. 6). How a plant cell is sensitized mechanically, is still unknown. However, it is majorly believed that plant cell wall and membrane interface has an important role to play in perception of mechanical stimulus^5^,^10^. Upon mechanical disturbance, cytoskeleton coordinates with and opens various stretch activated ion channels^5^,^10^. It has already been noted that SV affect cell wall and membrane microstructure, thereby resulting in an overall increase cell membrane tension^8^,^60^,^61^,^62^. Strong upregulation of *TCH4*, a cell wall modifying xyloglucan endotransglucosylase/hydrolase (XTH)^1^, was observed in microarray result. In response to SV, XTH could function as an important player for alteration of elasticity of cell wall. Thus, it can be hypothesized that SV is perceived at cell wall-cell membrane interface, which leads to the activation of membrane-bound mechanosensitive ion channels. Thereafter, signals are transduced into the cell in which calcium binding CMLs and various kinases play critical role. Sidewise, ROS are generated which act as signaling molecule for various complex metabolic processes. For keeping a check on toxic effects of ROS, various ROS scavenging enzymes are increased and/or activated. Kinases transduce the message downstream through phosphorylation/de-phosphorylation of different signaling proteins or transcription factors eventually resulting into gene expression. Huge changes in the transcriptome and proteome thus occur, which eventually affect several vital processes, like photosynthesis, glycolysis, amino acid metabolism and sulfur metabolism. These changes together with hormonal adjustments result in enhanced growth and defense.

Employing high throughput transcriptomic, proteomic and hormonal analyses, we revealed the global cellular changes taking place in a plant exposed to SV. Interestingly, our results go hand in hand with several previous reports on effect of SV in plants, providing strong scientific basis to this least explored area of plant biology. Indeed, there are many more facets of plant acoustics left to be explored in upcoming research.

## Methods

### Plant Materials and SV Treatment

*Arabidopsis thaliana* (Col-0 ecotype) seeds were placed in pots containing artificial soil (Punong, Korea) and stratified for 2 d in dark at 4 °C for homogenous germination. After stratification, pots were transferred to growth room and seedlings were allowed to grow under continuous light (~150 ± 10 μmol m^−2^ s^−1^) at 22 ± 1 °C for 20 d. Growing seedlings were supplemented with nutrients (Bio-nex, Korea) mixed in water at every 3 d interval. Sound level of growth room, as recorded by sound level meter TES-1350A (Pusung, Korea), was noted to be 75 ± 2 dB. Twenty-d-old Arabidopsis plants were transferred to sound-proof chamber and subjected to 5 different frequencies of SV (250, 500, 1000, 2000, and 3000 Hz) with constant amplitude (80 dB) separately for 1 h. Sound-proof chamber was customized by Korea Scientific Technique Industry (Korea) according to Jeong, *et al*.^12^. The sound level within the chamber was recorded to be 40 dB. The Adobe Audition version 3.0 software (USA) was used for generation of single frequency sound. To prevent the mechanical vibrations during the SV treatments, the speaker and plants were placed on different shelves. After SV treatment, plants were shifted to growth room and samples were harvested at requisite time points for transcriptomic, proteomic and hormonal analyses. For transcriptomic analysis, samples at 0, 0.5 and 6 h after SV treatments were harvested. Similarly, samples at 0, 1, 24 and 48 h were harvested for proteomic analysis. For hormonal analysis plants treated with 500 Hz (80 dB) were selected and samples were harvested at 6, 24 and 48 h after SV treatment. For a better representation of treatment method and sample harvesting time, a schematic diagram has been shown in Supplementary Fig. S1.

### qRT-PCR

Total RNA from Arabidopsis rosette was extracted with the RNeasy Plant Mini kit (Qiagen, USA) according to the manufacturer’s instruction. RNA samples were treated with DNase I (Qiagen) for removal of DNA contamination. cDNA was prepared with 1 μg RNA using GoScript Reverse Transcription system (Promega, USA) as per manufacturer’s instruction. cDNA was diluted by 10 fold before using as a template for qRT-PCR analysis. qRT-PCR was performed using Mx3000P qPCR system (Agilent, USA) and LF Taq qPCR SYBR Mix (LPS Solution, Korea). Primers were designed in Primer3 program of Biology WorkBench (http://workbench.sdsc.edu/) based on the following parameters: 23 to 27 nucleotides, T_m_ 60 °C ± 3 °C, product/amplicon size 200 to 250 bp. Primers used are listed in Supplementary Table S8. Prior to qRT-PCR, specificity of primers for each gene was checked by analyzing individual dissociation/melting curve. At1g13440 (*GAPDH*) encoding glyceraldehyde-3-phosphate dehydrogenase was used as an internal control. Threshold of 0.1 was set manually to obtain a threshold cycle (C_T_) value for each gene. C_T_ values for all genes of interest (C_T_._GOI_) were normalized to the C_T_ values of *GAPDH* (C_T_. _GAPDH_) for each replication [∆C_T_ = (C_T_._GOI_) − (C_T_. _GAPDH_)] as suggested by Schmittgen, *et al*.^63^. Relative transcript levels of each gene were calculated with respect to *GAPDH* (% relative expression to *GAPDH*) using 2^−∆CT^ value [2^−∆CT^ × 100] and plotted in graph^63^. Mean values were obtained from four biological replicates, and the standard errors are indicated by error bars.

### Transcriptomic Profiling

Global gene expression profiling was conducted using Affymetrix GeneChip^®^ Arabidopsis ATH1 Genome arrays (USA) and all data have been deposited in the GEO database (http://www.ncbi.nlm.nih.gov/geo/; accession number GSE68944). RNA was isolated as above and the sample was prepared according to manufacturer’s instruction. RNA quality was assessed by Agilent 2100 Bioanalyser using the RNA 6000 Nano Chip (Agilent) and quantity was determined by ND-1000 spectrophotometer (NanoDrop Technologies, USA). RNA (6 μg) was used per sample as input into the Affymetrix procedure as recommended in the protocol (http://www.affymetrix.com). Briefly, double-strand cDNA was prepared using oligo (dT) primer incorporating a T7 promoter. Amplified RNA (cRNA) was generated from the double-stranded cDNA template though an *in vitro* transcription reaction and purified with the Affymetrix sample cleanup module. Subsequently, amplified RNA was fragmented using 8 μl of 5× fragmentation buffer (Sample cleanup Module; Affymetrix). Fragmentation was checked on 1% agarose gel stained with ethidium bromide and hybridized to the gene containing over 22,500 probe sets, as described in the gene chip expression analysis technical manual (Affymetrix). After hybridization, the chips were stained and washed in a Genechip Fluidics Station 450 (Affymetrix) and scanned by using a Genechip Array scanner 3000 7G (Affymetrix).

After the final wash and staining step, the array was scanned using Affymetrix Model 3000 G7 scanner. Affymetrix Command Console (AGCC) software 1.1 was used for image data extraction. The raw.cel file generated through above procedure having expression intensity data was used for the next step. Expression data were generated by AGCC software version 1.1. For the normalization, Robust Multi-Average (RMA) algorithm implemented in AGCC software was used. To reduce noise for the significance analysis, probe sets that were not present by the MAS5 detection call in over 50% of the samples in at least one sample group were filtered out. To determine whether genes were differentially expressed between the two groups, an unpaired *t*-test was performed on the RMA expression values. A multiple testing correction^64^ was applied to the *P*-values of the *F*-statistics to adjust the false discovery rate. Genes with adjusted *F*-statistic *P*-values < 0.05 were extracted. Highly expressed genes that showed over 2-fold differences comparing the signal value of control and each test group, were selected for further study. Fold changes and *P*-values were obtained from three biological replicates. In order to classify the co-expression gene group which has gene with similar expression pattern, we performed hierarchical clustering in Multi Experiment Viewer (MEV) software 4.0 (http://www.tm4.org). The web-based tool, the Database for Annotation, Visualization, and Integrated Discovery (DAVID) was used to perform the biological interpretation of differentially expressed genes. Then, these genes were classified based on the information of gene function in GO analysis in the TAIR (http://www.arabidopsis.org) and Panther Ontology database (http://david.abcc.ncifcrf.gov/home.jsp).

### Proteomic Profiling

Protein extraction, 2-DGE, in-gel digestion, identification and functional classification were conducted as previously described by Kwon, *et al*.^51^. Briefly, protein was extracted from rosette using TCA/acetone/phenol extraction method and concentration was determined by 2D-Quant kit (GE Healthcare Life Sciences, USA). Subsequently, 2-DGE was performed by using 17-cm immobilized pH gradient (IPG) strip pH 5–8 in a Protean^®^ Isoelectric Focusing (IEF) cell (Bio-Rad, USA) and stained with silver. Imaging and analysis were done by GS-800 Imaging Densitometer (Bio-Rad) and PDQuest version 7.2.0 software (Bio-Rad), respectively. All experiments were performed with three replicates. Proteins showing statistically significant (*P* < 0.05, Student’s *t*-test) differential regulation (cut-off 1.5-fold) were chosen for further identification. In-gel trypsin digestion was conducted prior to protein identification by MALDI-TOF/MS (Applied Biosystem, USA). Peptide mass tolerance and fragment mass tolerance of the 78 selected proteins were set to 100 ppm. High confidence identifications had statistically significant search scores (>95% confidence) consistent with the protein’s experimental isoelectric point (*pl*) and molecular weight (MW). After that all seventy-eight identified proteins were classified based on GO and Mapman annotation of the plant proteome database (PPDB, http://ppdb.tc.cornell.edu/).

### Phytohormone Analysis

Hormone analysis was performed according to Pan *et al*.^65^. Ground tissue (50 mg) was used for hormone measurement. To separate out individual plant hormones in a mixture, each of the analytes and internal standards were subjected to chromatography on a C18 reversed-phase column (Agilent), which is followed by analysis via electrospray ionization (ESI)-MS/MS (Applied Biosystem). Elutes were monitored by a serious multiple reaction monitoring (MRM). IAA, GA_3_, and JA were procured from Sigma-Aldrich (USA), 2-*cis*, 4-trans-Abscisic acid-[^2^H_6_] ABA (d_6_-ABA) was procured from OlChemIm Ltd. (Czech) and SA from TOKYO Chemical Industry Co. (Japan). The stock solutions of these hormones were prepared at a concentration of 1 mg mL^−1^ in 100% methanol. A working internal standard (IS) solution was prepared by diluting the d-ABA and d-zeatin stock solution with methanol to a final concentration of 1 μg mL^−1^. For HPLC, a 1100 series liquid chromatography system (Agilent) was used, which is equipped with a degasser, pump, auto sampler and column oven. Chromatographic separations were performed on a SunfireTM C18 (2.1 × 10 mm) column (Waters, USA). The mobile phase was an isocratic mobile phase of 15:85 (v/v) 0.1% formic acid in water/0.1% formic acid in methanol, at a flow rate of 300 μL min^−1^. The column temperature and injection volume was 30 °C and 10 μL, respectively in all experiments. For linearity analysis a standard calibration curve for each hormone was prepared. Calibration curves were estimated using the ratio of ABA, GA_3_, IAA, JA, SA and zeatin area/IS area versus the ratio of ABA, GA_3_, IAA, JA, SA and zeatin/IS concentration. Each calibration curve was assayed individually by using 1/x weighted linear regression. The limit of detection (LOD) was estimated by using a signal-to-noise ratio of 3 and the limit of quantification (LOQ) was estimated by using a signal-to-noise of 10.

## Additional Information

**How to cite this article**: Ghosh, R. *et al*. Exposure to Sound Vibrations Lead to Transcriptomic, Proteomic and Hormonal Changes in Arabidopsis. *Sci. Rep.* **6**, 33370; doi: 10.1038/srep33370 (2016).

## Supplementary Material

Supplementary Information

Supplementary Table 1

## Figures and Tables

**Table 1:** List of genes showing fold change in microarray and confirmed by qRT-PCR.

Locus	Gene	Hertz					Function
		250	500	1000	2000	3000	
At1g76650	CML38	7.53**	12.14**	6.50**	6.54**	6.76**	calmodulin-like gene
At1g33720	CYP76C6	1.23	4.10**	2.72**	2.61**	3.12**	secondary metabolism
At1g01560	MPK11	2.87	5.67**	4.62**	3.10**	3.06**	cellular signaling
At3g50060	MYB77	2.67**	3.78**	2.07^*^	2.76**	2.14**	transcription factor
At1g76600	—	2.72**	4.04**	2.08*	2.34**	2.15**	unknown
At1g25400	—	2.96**	4.58**	2.48**	2.27^*^	2.72**	unknown
At3g07350	—	3.84**	5.36**	1.88	6.00**	4.80**	unknown
At3g27690	LHCB2	1.68	7.32**	1.04	6.58**	4.70**	photosynthesis
At2g20670	—	10.76**	15.80**	4.34	13.08**	9.36*	unknown
At1g21910	DREB26	7.39*	11.29*	8.03*	9.47*	8.10*	transcription factor
At2g44500	—	4.44	6.43*	4.90*	4.69	4.47	unknown
At2g40000	HSPRO2	4.43	7.62*	4.59	3.12	3.60	defense response
At1g13260	RAV1	4.38*	5.00*	4.24*	4.15*	4.01*	transcription factor
At1g70290	TPS8	5.61*	7.52*	4.01	5.68*	5.18	trehalose biosynthesis
At4g37610	BT5	5.05*	7.48**	3.74	3.60	3.57	BTB and TAZ domain protein
At1g80440	KMD1	6.59*	6.86*	3.72	5.48	3.97	phenylpropanoid metabolism
At5g22920	RZPF34	5.48	4.07	3.53	3.29	3.00	stomatal opening regulation

Numerical value represents the microarray fold changes. *P*-value ranges are marked by asterisks:

^**^P < 0.05,

^*^P < 0.1.
