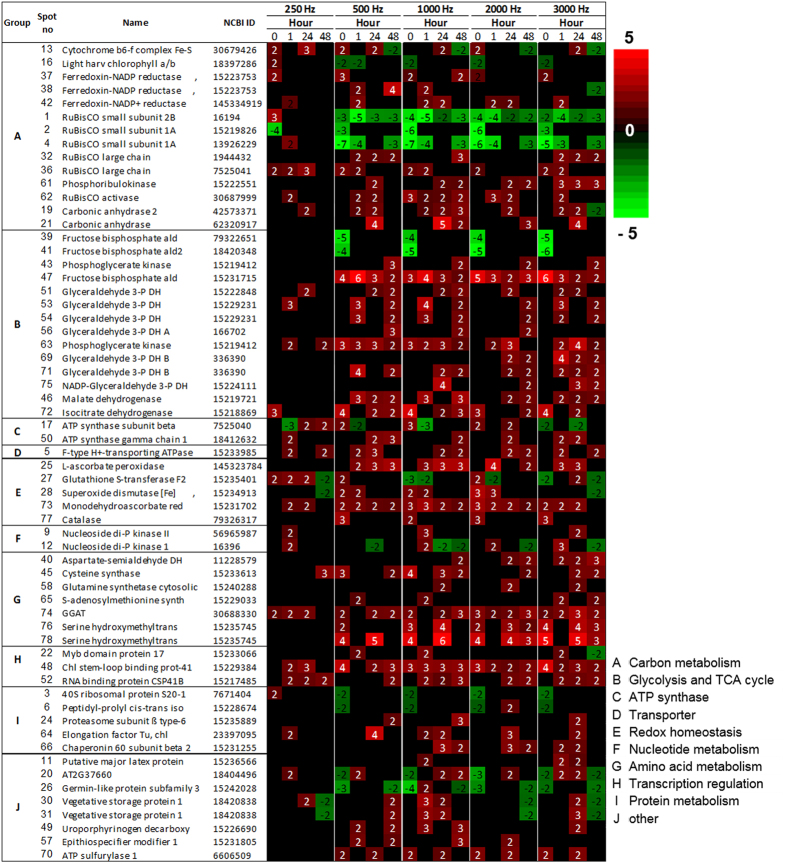# Corrigendum: Exposure to Sound Vibrations Lead to Transcriptomic, Proteomic and Hormonal Changes in Arabidopsis

**DOI:** 10.1038/srep37484

**Published:** 2016-11-24

**Authors:** Ritesh Ghosh, Ratnesh Chandra Mishra, Bosung Choi, Young Sang Kwon, Dong Won Bae, Soo-Chul Park, Mi-Jeong Jeong, Hanhong Bae

Scientific Reports 6: Article number: 3337010.1038/srep33370; published online: 09 26 2016; updated: 11 24 2016

This Article contains an incorrect version of Figure 4. The correct version of Figure 4 appears below as Figure 1.

## Figures and Tables

**Figure 1:**