# Supplementary material for: Exposure to Sound Vibrations Lead to Transcriptomic, Proteomic and Hormonal Changes in Arabidopsis
Source: Sci Rep. 2016 Sep 26;6:33370. doi: 10.1038/srep33370 (PMC5036088; doi:10.1038/srep33370)

Exposure to Sound Vibrations Lead to Transcriptomic, Proteomic and Hormonal Changes in Arabidopsis

Ritesh Ghosh[1], Ratnesh Chandra Mishra[1], Bosung Choi[1], Young Sang Kwon[2], Dong Won Bae[3], Soo-Chul Park[4], Mi-Jeong Jeong[4*], Hanhong Bae[1*]

[1] Department of Biotechnology, Yeungnam University, Gyeongsan 38541, Republic of Korea,

[2] Environmental Biology and Chemistry Center, Korea Institute of Toxicology, Jinju 52834, Republic of Korea

[3] Central Instrument Facility, Gyeongsang National University, Jinju 52828, Republic of Korea

[4] National Institute of Agricultural Sciences, Rural Development Administration, Wanju 55365, Republic of Korea

Supplementary Table S2. Showing details of selected genes which were used for microarray heatmap preparation in Fig. 2.

TAIR ID	Name	Involved in
At4g29780	--	Unknown
At4g24570	DIC2	Mitochondrial transport; dicarboxylic acid transmembrane transporter activity
At3g04640	--	Unknown
At5g57560	TCH4	Cell wall biogenesis; xyloglucan metabolic process; response to auxin, BR, cold, heat, mechanical stimulus
At3g55980	SZF1	Regulation of transcription; response to chitin
At1g76600	--	Unknown
At1g35140	EXL1	Growth; response to hypoxia
At1g76650	CML38	Response to wounding; calcium ion binding
At1g01560	MPK11	Response to ABA; signal transduction
At1g16130	WAKL2	Protein autophosphorylation; cell surface receptor signaling pathway
At4g00970	CRK41	Defense response, protein phosphorylation
At3g50060	MYB77	Lateral root development, regulation of transcription, response to chitin, ET, SA stimulus
At1g21910	DREB26	Cellular response to freezing, heat; positive regulation of transcription; response to JA, SA stimulus; ethylene-activated signaling pathway
At4g17500	ERF1	Ethylene-activated signaling pathway; regulation of transcription; defense response; cell division
At5g67300	MYB44	Defense response to bacterium, fungus; regulation of JA, SA mediated signaling; regulation of transcription; response to ABA, auxin, ET, gibberellin, SA stimulus; response to cadmium ion, chitin, salt stress, water deprivation
At5g47220	ERF2	Cell division, ET-activated signaling, induced systemic resistance; JA mediated signaling; regulation of transcription; response to chitin
At5g49520	WRKY48	Defense response to bacterium; positive regulation of transcription; response to chitin
At1g33760	--	Regulation of transcription; ET-activated signaling pathway
At5g22380	ANAC090	Multicellular organismal development; regulation of transcription
At2g25900	ATTZF1	Regulation of transcription; DNA-dependent
At4g29190	ATOZF2	Regulation of transcription
At3g07340	CIB3	Regulation of transcription
At1g13260	RAV1	Lateral root and leaf development; negative regulation of transcription; response to BR stimulus; ET-activated signaling pathway
At1g03850	GRXS13	Cell redox homeostasis; defense response to fungus; response to cytokinin, photo oxidative stress
At4g21990	ATAPR3	Cell redox homeostasis; sulfate assimilation; cysteine biosynthetic process
At3g62950	--	Cell redox homeostasis
At4g04610	ATAPR1	Cell redox homeostasis; sulfate assimilation; cysteine biosynthetic process
At4g37370	CYP81D8	Rsponse to karrikin, secondary metabolite biosynthetic process
At1g33720	CYP76C6	Secondary metabolite biosynthetic process

Supplementary Table S2 continued.

TAIR ID	Name	Involved in
At3g27690	LHCB2	Photosynthesis; response to blue, far red, red light
At3g28340	GATL10	Carbohydrate biosynthesis; pectin biosynthetic process, cell wall organization, response to oxidative stress
At5g42250	--	Oxidation-reduction process
At1g70290	TPS8	Trehalose biosynthetic process
At4g08950	EXO	Response to BR stimulus
At4g09760	CEK3	Phosphorylation, choline kinase activity
At1g80440	--	Negative regulation of cytokinin-activated signaling, regulation of phenylpropanoid metabolic process
At2g34600	JAZ7	Defense response; regulation of JA mediated signaling; response to wounding and chitin
At5g06320	NHL3	Defense response to bacterium, virus; response to salicylic acid, wounding
At5g66070	--	Defense response; response to chitin
At4g33920	APD5	Protein dephosphorylation
At2g40000	HSPRO2	Defense response to bacterium; response to oxidative stress, SA stimulus
At2g41640	--	Unknown
At3g15450	--	Unknown
At4g37610	BT5	Regulation of transcription; response to chitin, cold, hydrogen peroxide; response to auxin, SA stimulus; protein ubiquitination
At1g25400	--	Unknown
At5g22920	RZPF34	Regulation of stomatal opening
At2g27660	--	Unknown
At2g21210	SAUR6	Response to auxin, chitin
At1g04770	--	Unknown
At4g11300	--	Unknown
At2g20670	--	Unknown
At3g07350	--	Unknown
At4g02075	--	Unknown
At5g60680	--	Unknown
At2g44500	--	Unknown
At4g19430	--	Unknown
At5g06790	--	Unknown
At1g48330	--	Unknown
At4g16447	--	Unknown

Supplementary Table S3. Statistical analysis (Duncan's multiple range test, DMRT) of qPCR results. The different letters in a time point indicate significant differences among each other with $P < 0.05$ as determined by DMRT.

Genes	0 h						0.5 h						6 h					
	Con	Hertz					Con	Hertz					Con	Hertz				
		250	500	1000	2000	3000		250	500	1000	2000	3000		250	500	1000	2000	3000
CML38	B	B	A	B	B	B	A	A	A	A	A	A	C	C	C	C	B	A
CYP76C6	B	B	A	B	B	B	B	B	B	B	A	B	BC	C	ABC	C	AB	A
MPK11	B	B	A	AB	B	B	A	A	A	A	A	A	A	A	A	A	A	A
MYB77	B	AB	A	B	B	B	A	AB	B	B	A	AB	B	B	B	B	A	A
At1g76600	B	B	A	B	B	B	A	B	B	B	AB	B	C	C	C	C	B	A
At1g25400	C	B	A	BC	BC	BC	A	B	AB	B	AB	B	AB	C	C	BC	A	AB
At3g07350	B	AB	A	B	A	AB	AB	BC	BC	C	AB	A	B	B	B	B	A	A
LHCB2	B	B	A	B	A	B	AB	B	B	B	A	AB	AB	B	AB	AB	A	A
At2g20670	B	AB	A	B	A	B	AB	BC	BC	C	A	AB	B	B	B	B	A	A
DREB26	C	AB	A	BC	AB	BC	A	A	A	A	A	A	B	B	B	B	A	A
At2g44500	C	A	A	AB	BC	BC	A	BC	BC	BC	AB	C	B	B	B	B	A	A
HSPRO2	B	B	A	B	B	B	A	C	BC	BC	AB	C	B	B	B	B	A	A
RAV1	C	AB	A	AB	AB	BC	B	B	AB	AB	A	B	BC	BC	B	C	A	A
TPS8	C	AB	A	BC	ABC	BC	A	B	B	B	B	B	B	B	B	B	A	A
BT5	C	AB	A	BC	C	C	AB	BC	BC	C	A	BC	B	B	B	B	A	A
KMD1	C	AB	A	BC	ABC	C	A	AB	A	BC	A	C	BC	C	BC	C	A	AB
RZPF34	B	A	AB	B	B	B	AB	ABC	A	BC	C	BC	C	C	C	C	B	A

Supplementary Table S4. List of differentially expressed proteins which were identified by MALDI-TOF analysis including theoretical MW (Da), pI values, ratio of matched peptides/detected peptides and sequence coverage.

[a] Numbers correspond to the 2-DE gels shown in Supplementary Fig. S3.
[b] Theoretical MW (Da) and pI values.
[c] Ratio of matched peptides/detected peptides.
[d] Sequence coverage.

No[a]	Protein	ID(NCBI)	MW/pI, theor.[b]	MP/TP[c]	SC [d] (%)
1	RuBisCO small subunit 2B	16194	20303/7.59	10/18	54
2	RuBisCO small subunit 1A	15219826	20203/7.59	12/27	48
3	40S ribosomal protein S20-1	7671404	13101/9.78	6/11	31
4	RuBisCO small subunit 1A, chloroplastic	13926229	14689/5.69	8/12	49
5	F-type H^+-transporting ATPase subunit delta	15233985	25653/9.04	5/10	27
6	Peptidyl-prolyl cis-trans isomerase CYP20-3	15228674	28190/8.83	4/10	14
7	Peptidyl-prolyl cis-trans isomerase CYP20-3	15228674	28190/8.83	10/18	34
8	Eukaryotic translation initiation factor 5A-2	9295717	23781/6.49	4/8	18
9	Nucleoside diphosphate kinase II	56965987	17049/5.54	7/12	43
10	Nucleoside diphosphate kinase Ia	3063661	16962/5.54	4/8	19
11	Putative major latex protein	15236566	17507/5.91	5/11	19
12	Nucleoside diphosphate kinase 1	16396	16229/7.03	6/11	38
13	Cytochrome b6-f complex iron-sulfur subunit	30679426	22518/8.58	5/12	28
14	2-Cys peroxiredoxin BAS1	15229806	29074/6.92	6/11	38
15	Ribose 5-phosphate isomerase A	15229349	29287/5.72	4/11	27
16	Light harvesting Chlorophyll a/b binding protein	18397286	28209/5.29	4/9	13
17	ATP synthase subunit beta	7525040	53900/5.38	11/28	26
18	Oxygen-evolving enhancer protein 1-2	15230324	34998/5.92	4/8	14
19	Carbonic anhydrase 2, chloroplastic	42573371	28326/5.36	7/13	26
20	AT2G37660	18404496	34858/8.37	7/15	23
21	Carbonic anhydrase	62320917	28166/5.29	6/10	30
22	Myb domain protein 17	15233066	33269/6.46	5/5	12
23	Glutathione S-transferase DHAR2	15222163	23392/5.79	5/11	25
24	Proteasome subunit beta type-6	15235889	25136/5.31	6/11	27
25	L-ascorbate peroxidase	145323784	27503/5.85	8/13	32
26	Germin-like protein subfamily 3	15242028	21822/6.26	5.12	34
27	Glutathione S-transferase F2	15235401	24114/5.92	10/16	50
28	Superoxide dismutase [Fe], chloroplastic	15234913	23776/6.06	7/12	22
29	Glutathione S-transferase F9	15224581	24131/6.17	7/14	47
30	Vegetative storage protein 1	18420838	30243/5.49	5/11	22
31	Vegetative storage protein 1	18420838	30243/5.49	4/7	13
32	Ribulose bisphosphate carboxylase large chain	1944432	47609/6.12	5/9	17
33	Ribulose 1,5-bisphosphate carboxylase /oxygenase large chain	27752799	47464/6.12	6/14	15
34	AT5G02240	18413869	27086/6.19	5/8	18
35	V-type proton ATPase subunit E1	1143394	26054/6.04	12/19	40
36	Ribulose bisphosphate carboxylase large chain	7525041	52922/5.88	7/11	16
37	Ferredoxin--NADP reductase, leaf isozyme 2	15223753	41142/8.51	7/10	18
38	Ferredoxin--NADP reductase, leaf isozyme 2	15223753	41142/8.51	6/12	18
39	Fructose-bisphosphate aldolase	79322651	41781/5.94	4/6	12
40	Aspartate-semialdehyde dehydrogenase	11228579	36620/5.39	5/8	10
41	Probable fructose-bisphosphate aldolase 2	18420348	42961/6.78	8/12	27
42	Ferredoxin--NADP+ reductase	145334919	29665/5.91	7/15	21

Supplementary Table S4 continued.

No[a]	Protein	ID(NCBI)	MW/pI, theor.[b]	MP/TP[c]	SC [d] (%)
43	Phosphoglycerate kinase	15219412	42105/5.49	7/9	18
44	Phosphoglycerate kinase	15219412	42105/5.49	5/9	17
45	Cysteine synthase	15233613	33784/5.91	5/10	12
46	Malate dehydrogenase	15219721	35548/6.11	9/11	29
47	Fructose-bisphosphate aldolase	15231715	38516/6.05	12/18	34
48	Chloroplast stem-loop binding protein-41	15229384	43903/8.54	7/16	14
49	Uroporphyrinogen decarboxylase 2	15226690	43553/8.29	23/26	38
50	ATP synthase gamma chain	18412632	40886/8.13	6/9	13
51	Glyceraldehyde 3-phosphate dehydrogenase	15222848	36890/6.67	20/23	51
52	RNA binding protein CSP41B	15217485	42593/8.19	13/21	39
53	Glyceraldehyde-3-phosphate dehydrogenase	15229231	36891/6.62	7/11	22
54	Glyceraldehyde-3-phosphate dehydrogenase	15229231	36891/6.62	11/23	30
55	Hydroxypyruvate reductase	15220620	42221/6.68	18/18	43
56	Glyceraldehyde-3-phosphate dehydrogenase A	166702	37652/7.00	10/27	23
57	Epithiospecifier modifier 1	15231805	44032/7.59	6/10	27
58	Glutamine synthetase cytosolic isozyme 1-1	15240288	39090/5.28	7/8	13
59	Glutamine synthetase	15238559	47381/6.43	8/19	30
60	Glutamine synthetase	15238559	47381/6.43	10/18	31
61	Phosphoribulokinase	15222551	44436/5.71	12/18	31
62	Ribulose bisphosphate carboxylase /oxygenase activase	30687999	48469/7.55	14/24	31
63	Phosphoglycerate kinase	15219412	42105/5.49	5/8	12
64	Elongation factor Tu, chloroplastic	23397095	51624/5.84	12/24	15
65	S-adenosylmethionine synthase 4	15229033	42769/5.51	12/15	33
66	Chaperonin 60 subunit beta 2	15231255	63302/5.60	15/18	22
67	Myrosinase 1	30690085	61094/5.61	17/20	33
68	Phosphoglycerate dehydrogenase-like protein	21536501	63271/6.32	8/14	14
69	Glyceraldehyde-3-phosphate dehydrogenase B	336390	42769/5.60	6/9	15
70	ATP sulfurylase 1	6606509	51412/6.30	7/12	14
71	Glyceraldehyde-3-phosphate dehydrogenase B	336390	42769/5.60	10/14	23
72	Isocitrate dehydrogenase	15218869	45717/6.13	13/18	23
73	Monodehydroascorbate reductase	15231702	46458/6.41	10/14	25
74	Glutamate-glyoxylate aminotransferase 1	30688330	53267/6.49	18/29	30
75	NADP-dependent glyceraldehyde-3-phosphate dehydrogenase	15224111	53026/6.23	5/9	11
76	Serine hydroxy methyltransferase	15235745	57364/8.13	10/14	10
77	Catalase	79326317	54960/6.34	12/18	29
78	Serine hydroxy methyltransferase	15235745	57364/8.13	11/15	16

Supplementary Table S5. Details of differentially expressed proteins which were identified by MS/MS including peptide hit and peptide sequence.

[a] Score is the protein score based on combined mass and mass/mass spectrums.

[b] Peptide hit is the unique number of MS/MS spectrums which match to the trypsin peptide.

No. correspond to the 2-DE gels shown in Supplementary Fig. S3.

Protein	ID(NCBI)	SC (%)	Score [a]	Peptide hit[b]	Peptides identified
RuBisCO small subunit 2B	16194	59	453	7	K.EVDYLLR.N<br>R.YWTMWK.L<br>R.IIGFDNTR.Q<br>K.EYPGAFIR.I<br>R.EHGNTPGYYDGR.Y<br>K.LPLFGCTDSAQVLK.E<br>K.WIPCVEFELEHGFVYR.E
RuBisCO small subunit 1A	15219826	54	346	7	K.EVDYLLR.N<br>R.YWTMWK.L<br>R.IIGFDNTR.Q<br>K.EYPGAFIR.I<br>R.EHGNTPGYYDGR.Y<br>K.LPLFGCTDSAQVLK.E<br>K.WIPCVEFELEHGFVYR.E
RuBisCO small subunit 1A, chloroplastic	13926229	51	160	4	K.EVDYLIR.N<br>K.EYPNAFIR.I<br>R.EHGNSPGYYDGR.Y<br>K.WIPCVEFELEHGFVYR.E
F-type H+-transporting ATPase subunit delta	15233985	30	130	3	K.LTDTQLAEVR.S<br>K.TVIDASLVAGFTIR.Y<br>K.SSSLQSHTSNFLNVLVDANR.I
Peptidyl-prolyl cis-trans isomerase CYP20-3	15228674	14	57	1	K.VYFDVEIGGEVAGR.I
Peptidyl-prolyl cis-trans isomerase CYP20-3	15228674	18	59	2	R.TLESQETR.A<br>K.VYFDVEIGGEVAGR.I
Eukaryotic translation initiation factor 5A-2	9295717	24	61	1	K.CHFVAIDIFTAK.K
Nucleoside diphosphate kinase II	56965987	47	187	5	R.EIGLWFK.E<br>K.WDSALATWLR.E<br>K.TDPLQAEPGTIR.G<br>K.WDSALATWLRE.-<br>-.SMEDVEETYIMVKPDGIQR.G
Putative major latex protein	15236566	28	100	2	R.GLDGHVMEHLK.V<br>K.VFDIIYEFIPK.S
Cytochrome b6-f complex iron-sulfur subunit	30679426	31	135	3	K.VLFVPWVETDFR.T<br>K.FLCPCHGSQYNAQGR.V<br>R.GPAPLSLALAHADIDEAGK.V
2-Cys peroxiredoxin BAS1	15229806	38	242	4	K.SFGVLIHDQGIALR.G<br>K.APDFEAEAVFDQEFIK.V<br>K.LNTEVLGVSVDSVFSHLAWVQTDR.K<br>R.TLQALQYIQENPDEVCPAGWKPGEK.S

Supplementary Table S5 continued.

Protein	ID(NCBI)	SC (%)	Score [a]	Peptide hit[b]	Peptides identified
Ribose 5-phosphate isomerase A	15229349	30	123	2	R.SLGIPLVGLDTHPR.I<br>K.FQGVVEHGLFLGMATSVIIAGK.N
Light harvesting Chlorophyll a/b binding protein	18397286	16	79	2	K.FGEAVWFK.A<br>R.WAMLGALGCVFPELLAR.N
ATP synthase subunit beta	7525040	22	229	4	R.FVQAGSEVSALLGR.M<br>R.VGLTALTMAEYFR.D<br>R.DVNEQDVLLFIDNIFR.F<br>K.GIYPAVDPLDSTSTMLQPR.I
Oxygen-evolving enhancer protein 1-2	15230324	14	87	2	R.VPFLFTVK.Q<br>K.FKEEDGIDYAAVTVQLPGGER.V
Carbonic anhydrase 2, chloroplastic	42573371	26	168	3	K.YAGVGAAIEYAVLHLK.V<br>R.EAVNVSLANLLTYPFVR.E<br>R.VCPSHVLDFHPGDAFVVR.N
Carbonic anhydrase	62320917	25	158	2	R.EAVNVSLANLLTYPFVR.E<br>R.VCPSHVLDFQPGDAFVVR.N
Glutathione S-transferase DHAR2	15222163	25	86	2	K.IFGAFVTFLK.S<br>K.VAVGAPDVLGDCPFSQR.V
Germin-like protein subfamily 3	15242028	10	71	1	K.AAVTPAFAPAYAGINGLGVSLAR.L
Glutathione S-transferase F2	15235401	40	191	4	K.LFTERPR.V<br>K.VLDVYEAR.L<br>K.LAFEQIFK.S<br>R.NPFGQVPAFEDGDLK.L
Superoxide dismutase [Fe], chloroplastic	15234913	11	64	3	K.QTLEFHWGK.H<br>K.TFMTNLVSWEAVSAR.L<br>K.TFMTNLVSWEAVSAR.L + Oxidation (M)
Glutathione S-transferase F9	15224581	47	157	3	K.VYGPHFASPK.R<br>K.LAGVLDVYEAHLSK.S<br>K.YLAGDFVSLADLAHLPFTDYLVGPIGK.A
Vegetative storage protein 1	18420838	25	113	3	K.GYNIVGNIGDQWADLVEDTPGR.V<br>R.SWHLGVETSNIINFDTVPANCK.A<br>K.KGYNIVGNIGDQWADLVEDTPGR.V
Vegetative storage protein 1	18420838	13	73	1	K.GYNIVGNIGDQWADLVEDTPGR.V
V-type proton ATPase subunit E1	1143394	40	124	3	K.IFLPPPPK.S<br>R.LKEPSVLLR.C<br>K.DLIVQCLLR.L
Ribulose bisphosphate carboxylase large chain	7525041	16	66	2	R.ESTLGFVDLLR.D<br>R.FLFCAEAIYK.S

Supplementary Table S5 continued.

Protein	ID(NCBI)	SC (%)	Score [a]	Peptide hit[b]	Peptides identified
Ferredoxin--NADP reductase, leaf isozyme 2	15223753	18	176	4	R.MAQYAAELWELLK.K<br>K.DPNATVIMLATGTGIAPFR.S<br>K.DPNATVIMLATGTGIAPFR.S + Oxidation (M)<br>K.ITADDAPGETWHMVFSHQGEIPYR.E
Ferredoxin--NADP reductase, leaf isozyme 2	15223753	18	155	4	R.MAQYAAELWELLK.K<br>K.DPNATVIMLATGTGIAPFR.S<br>K.DPNATVIMLATGTGIAPFR.S + Oxidation (M)<br>K.ITADDAPGETWHMVFSHQGEIPYR.E
Fructose-bisphosphate aldolase	79322651	12	153	3	K.AAQDILLAR.A<br>R.TAAYYQQGAR.F<br>K.GLVPLVGSYDESWCQGLDGLASR.T
Probable fructose-bisphosphate aldolase 2	18420348	27	218	5	R.TAAYYQQGAR.F<br>K.MVDVLVEQNIVPGIK.V<br>K.TWGGRPENVNAAQTTLLAR.A<br>K.GLVPLVGSNNESWCQGLDGLSSR.T<br>R.YAAISQDSGLVPIVEPEILLDGEHDIDR.T
Ferredoxin--NADP+ reductase	145334919	21	94	1	K.DPNATIIMLGTGTGIAPFR.S
Phosphoglycerate kinase	15219412	18	80	2	K.LVAGLPEGGVLLLENVR.F<br>K.LAALADVYVNDAFGTAHR.A
Phosphoglycerate kinase	15219412	17	68	1	K.LVAGLPEGGVLLLENVR.F
Malate dehydrogenase	15219721	29	61	1	R.VLVTGAAGQIGYALVPMIAR.G
Fructose-bisphosphate aldolase	15231715	34	254	5	K.KPWSLSFSFGR.A<br>K.VSPEVIAEHTVR.A<br>K.FADELIANAAYIGTPGK.G<br>K.IGENEPSEHSIHENAYGLAR.Y<br>R.TVPAAVPAIVFLSGGQSEEEATR.N
Uroporphyrinogen decarboxylase 2	15226690	38	419	7	K.GEVVDRPPVWLMR.Q<br>R.EFVPEESVPYVGEALR.R<br>K.GPIIFNPPQSAADVAQVR.E<br>R.TGVDVVSLDWTVDMAEGR.D<br>K.GKGPIIFNPPQSAADVAQVR.E<br>K.VGTPEENVAHFFEVAQEIR.Y<br>K.QTHPNLPLILYASGSGGLLER.L
ATP synthase gamma chain 1	18412632	13	92	2	R.GLCGGFNNFIIK.K<br>R.ALQESLASELAAR.M
Glyceraldehyde 3-phosphate dehydrogenase	15222848	51	356	5	R.VPTVDVSVVDLTVR.L<br>K.TLLFGEKPVTVFGIR.N<br>K.LVSWYDNEWGYSSR.V<br>R.FGIVEGLMTTVHSITATQK.T<br>K.GILGYTEDDVVSTDFVGDNR.S

Supplementary Table S5 continued.

Protein	ID(NCBI)	SC (%)	Score [a]	Peptide hit[b]	Peptides identified
RNA binding protein CSP41B	15217485	39	242	6	R.FIGLFLSR.I<br>K.EGHQVTLFTR.G<br>K.DLATAFLNVLGNEK.A<br>K.AGGFPEPEIVHYNPK.E<br>K.AGRPIPVPNSGIQISQLGHVK.D<br>K.HVLGWKPEFDLVEGLTDSYNLDFGR.G
Glyceraldehyde-3-phosphate dehydrogenase	15229231	30	196	3	R.VPTVDVSVVDLTVR.L<br>K.TLLFGEKPVTVFGIR.N<br>K.LVSWYDNEWGYSSR.V
Hydroxypyruvate reductase	15220620	43	349	7	R.IVEADEFMR.G<br>K.FVTAYGQFLK.A<br>R.WINLLVDQGCR.V<br>R.GPVIDEAALVEHLK.E<br>K.MNLIYFDLYQSTR.L<br>R.GGLYEGWLPHLFVGNLLK.G<br>K.YGIAVGNTPGVLTETTAELAASLSLAAA R.R
Glyceraldehyde-3-phosphate dehydrogenase A	166702	23	161	3	K.TFAEEVNAAFR.D<br>K.VIAWYDNEWGYSQR.V<br>K.ELGIDIVIEGTGVFVDR.E
Glutamine synthetase cytosolic isozyme 1-1	15240288	13	132	2	K.IIAEYIWVGGSGMDMR.S<br>R.LTGHHETADINTFLWGVANR.G
Glutamine synthetase	15238559	25	231	4	R.IIAEYIWIGGSGIDLR.S<br>K.HETASIDQFSWGVANR.G<br>R.GGNNILVICDTWTPAGEPIPTNKR.A<br>K.WNYDGSSTGQAPGEDSEVILYPQAIFR .D
Glutamine synthetase	15238559	25	280	5	K.AILNLSLR.H<br>R.IIAEYIWIGGSGIDLR.S<br>K.HETASIDQFSWGVANR.G<br>R.GGNNILVICDTWTPAGEPIPTNKR.A<br>K.WNYDGSSTGQAPGEDSEVILYPQAIFR .D
Phosphoribulokinase	15222551	31	182	4	K.ILVIEGLHPMFDER.V<br>K.ILVIEGLHPMFDER.V + Oxidation (M)<br>R.VRDLLDFSIYLDISNEVK.F<br>K.YFSPVYLFDEGSTISWIPCGR.K
Ribulose bisphosphate carboxylase /oxygenase activase	30687999	21	217	5	K.FYWAPTR.E<br>K.FYWAPTREDR.I<br>K.MCCLFINDLDAGAGR.M<br>R.VPIICTGNDFSTLYAPLIR.D<br>K.IKDEDIVTLVDQFPGQSIDFFGALR.A
Phosphoglycerate kinase	15219412	12	149	2	K.LVAGLPEGGVLLLENVR.F<br>K.LAALADVYVNDAFGTAHR.A
Elongation factor Tu, chloroplastic	23397095	15	146	3	K.ILDEALAGDNVGLLLR.G<br>K.IVVELIVPVACEQGMR.F<br>R.QTELPFLLAVEDVFSITGR.G

Supplementary Table S5 continued.

Protein	ID(NCBI)	SC (%)	Score [a]	Peptide hit[b]	Peptides identified
S-adenosylmethionine synthase 4	15229033	28	115	4	K.TIFHLNPSGR.F<br>R.FVIGGPHGDAGLTGR.K<br>K.ESFDFRPGMISINLDLK.R<br>R.RVIVQVSYAIGVPEPLSVFVDSYGTGK.I
Chaperonin 60 subunit beta 2	15231255	18	216	4	R.DLVGVLEDAIR.G<br>K.AAVEEGIVVGGGCTLLR.L<br>K.SQYLDDIAILTGATVIR.E<br>K.SAENNLYVVEGMQFDR.G
Myrosinase 1	30690085	30	357	5	R.GLNVWDSFTHR.F<br>K.NWITINQLYTVPTR.G<br>K.GFIFGVASSAYQVEGGR.G<br>K.GYFAWSLGDNYEFCNGFTVR.F<br>K.TIVDDFKDYADLCFELFGDR.V
Glyceraldehyde-3-phosphate dehydrogenase B	336390	15	71	1	K.VVAWYDNEWGYSQR.V
ATP sulfurylase 1	6606509	14	159	4	R.NADAVFAFQLR.N<br>R.INAGANFYIVGR.D<br>R.ESEFLQTLHFNSLR.L<br>R.LNILPFR.V
Glyceraldehyde-3-phosphate dehydrogenase B	336390	25	101	1	K.VVAWYDNEWGYSQR.V
Isocitrate dehydrogenase	15218869	22	254	7	R.HAFGDQYR.A<br>R.NILNGTVFR.E<br>K.YFDLGLPHR.D<br>K.YDAAGIWYEHR.L<br>K.LVPGWTKPICIGR.H<br>K.SKYDAAGIWYEHR.L<br>K.GGETSTNSIASIFAWTR.G
Monodehydroascorbate reductase	15231702	25	168	5	R.FGAYWVQGGK.V<br>R.RVEHVDHSR.K<br>K.YQTLIIATGSTVLR.L<br>K.AVVVGGGYIGLELSAVLR.I<br>K.AAEGGAAVEEYDYLPFFYSR.S
Glutamate-glyoxylate aminotransferase 1	30688330	30	215	6	K.GYWGECGQR.G<br>K.GVMQILNCVIR.G<br>R.GGYFEMTNLPPR.V<br>K.NVVCNFTEGAMYSFPQIR.L<br>K.IIFTNVGNPHALGQKPLTFPR.Q<br>K.LVLLGDEVYQQNIYQDERPFISSK.K
Catalase	79326317	27	272	3	K.TWPEDILPLQPVGR.M<br>R.EGNFDLVGNNFPVFFIR.D<br>K.GFFEVTHDISNLTCADFLR.A

Supplementary Table S6. Showing proteomic fold change and *P*-value at 0, 1, 24 and 48 h after sound vibration treatments. [a] indicates the numbers correspond to the 2-DE gels shown in Supplementary Fig. S3.

No[a]	Protein	250 Hz		500 Hz		1000 Hz		2000 Hz		3000 Hz	
		Fold change	*P* value	Fold change	*P* value	Fold change	*P* value	Fold change	*P* value	Fold change	*P* value
**0 h**											
1	RuBisCO small subunit 2B	3.17	*0.001*	-2.76	*0.000*	-4.39	*0.000*	-3.75	*0.000*	-2.38	*0.001*
2	RuBisCO small subunit 1A	-4.07	*0.000*	-3.48	*0.000*	-6.15	*0.000*	-6	*0.000*	-3.48	*0.000*
3	40S ribosomal protein S20-1	1.9	*0.042*	-1.97	*0.011*	-1.74	*0.027*	-2.28	*0.012*	-2.15	*0.016*
4	RuBisCO small subunit 1A, chloroplastic			-7.26	*0.000*	-7.26	*0.000*	-6.35	*0.000*	-5.45	*0.000*
6	Peptidyl-prolyl cis-trans isomerase CYP20-3			-1.85	*0.025*	-1.68	*0.028*	-1.76	*0.032*	-1.68	*0.021*
13	Cytochrome b6-f complex iron-sulfur subunit	2.04	*0.003*	2.25	*0.004*			2.17	*0.000*		
16	Light harvesting chlorophyll a/b binding protein	1.82	*0.006*	-1.73	*0.013*	-1.79	*0.018*	-1.86	*0.003*	-1.58	*0.012*
17	ATP synthase subunit beta			2.13	*0.004*	2.52	*0.000*	2.13	*0.004*	-1.89	*0.010*
20	AT2G37660			-1.95	*0.001*	-2.2	*0.000*	-3.11	*0.000*	-1.99	*0.001*
26	Germin-like protein subfamily 3			-2.87	*0.000*	-3.91	*0.000*	-2.68	*0.000*	-2.11	*0.001*
27	Glutathione S-transferase F2	2.08	*0.022*	2	*0.006*	-2.59	*0.000*	2.41	*0.000*	-2.41	*0.007*
28	Superoxide dismutase [Fe], chloroplastic			2.18	*0.010*	2.36	*0.017*	3.45	*0.003*		
36	Ribulose bisphosphate carboxylase large chain	1.73	*0.096*	2.27	*0.039*	1.91	*0.017*	1.64	*0.046*	2	*0.021*
37	Ferredoxin--NADP reductase, leaf isozyme 2	2.46	*0.020*	2.63	*0.018*	1.66	*0.083*	1.85	*0.030*	1.9	*0.031*
39	Fructose-bisphosphate aldolase			-4.51	*0.000*	-3.81	*0.000*	-4.25	*0.000*	-4.73	*0.000*
41	Probable fructose-bisphosphate aldolase 2			-3.87	*0.000*	-4.8	*0.000*	-5.2	*0.000*	-6.22	*0.000*
45	Cysteine synthase			2.73	*0.001*	3.58	*0.001*				
47	Fructose-bisphosphate aldolase			3.5	*0.006*	3.25	*0.017*	4.5	*0.003*	5.63	*0.002*

Supplementary Table S6 continued.

No[a]	Protein	250 Hz		500 Hz		1000 Hz		2000 Hz		3000 Hz	
		Fold change	*P* value	Fold change	*P* value	Fold change	*P* value	Fold change	*P* value	Fold change	*P* value
**0 h**											
48	Chloroplast stem-loop binding protein-41			3.76	*0.000*	2.94	*0.002*	3.26	*0.001*	4.28	*0.000*
62	Ribulose bisphosphate carboxylase /oxygenase activase					2.89	*0.007*				
63	Phosphoglycerate kinase			3.22	*0.000*	2.71	*0.001*				
70	ATP sulfurylase 1			1.86	*0.046*	2.28	*0.032*	2.47	*0.019*	2.44	*0.010*
72	Isocitrate dehydrogenase	3.04	*0.000*	3.6	*0.009*	4.25	*0.005*	2.88	*0.001*	3.71	*0.001*
73	Monodehydroascorbate reductase			2.4	*0.004*	2.58	*0.003*	3.15	*0.003*	2.24	*0.005*
74	Glutamate-glyoxylate aminotransferase 1	1.86	*0.006*	1.91	*0.003*	2.28	*0.003*	2.52	*0.000*	1.92	*0.004*
76	Serine hydroxymethyltransferase			2.06	*0.017*	3.12	*0.006*	2.47	*0.013*	3.53	*0.003*
77	Catalase			3.14	*0.003*	2.29	*0.017*	2.57	*0.039*	2.86	*0.002*
78	Serine hydroxymethyltransferase			3.63	*0.001*	4.44	*0.001*	3.97	*0.002*	4.81	*0.001*
**1 h**											
1	RuBisCO small subunit 2B			-4.86	*0.000*	-5.28	*0.001*	-4.12	*0.000*	-3.8	*0.001*
4	RuBisCO small subunit 1A, chloroplastic	2.36	*0.000*	-3.53	*0.000*	-3.71	*0.000*	-4.24	*0.000*	-3.41	*0.000*
5	F-type H+-transporting ATPase subunit delta	1.95	*0.017*	2.02	*0.003*					2.33	*0.002*
7	Peptidyl-prolyl cis-trans isomerase CYP20-3	1.97	*0.000*								
9	Nucleoside diphosphate kinase II	1.7	*0.067*			2.5	*0.021*				
11	Putative major latex protein					1.81	*0.001*			2.46	*0.000*
12	Nucleoside diphosphate kinase 1	2.17	*0.000*			2.19	*0.000*	1.81	*0.015*	2.82	*0.000*
14	2-Cys peroxiredoxin BAS1									4.67	*0.002*
15	Ribose 5-phosphate isomerase A	-1.67	*0.005*								
16	Light harvesting chlorophyll a/b binding protein			-1.86	*0.017*						
17	ATP synthase subunit beta	-2.66	*0.014*	-1.55	*0.081*	-3.1	*0.012*				

Supplementary Table S6 continued.

No[a]	Protein	250 Hz		500 Hz		1000 Hz		2000 Hz		3000 Hz	
		Fold change	*P* value	Fold change	*P* value	Fold change	*P* value	Fold change	*P* value	Fold change	*P* value
	**1 h**										
18	Oxygen-evolving enhancer protein 1-2			-1.81	*0.010*						
19	Carbonic anhydrase 2, chloroplastic			1.79	*0.001*	1.64	*0.032*			2.01	*0.005*
20	AT2G37660	1.81	*0.001*	2.14	*0.000*					2.36	*0.000*
22	Myb domain protein 17			1.99	*0.000*	2.39	*0.000*			1.88	*0.001*
25	L-ascorbate peroxidase			2.42	*0.007*	2.58	*0.013*	3.92	*0.002*	2.83	*0.001*
26	Germin-like protein subfamily 3					1.89	*0.001*				
27	Glutathione S-transferase F2	2.38	*0.003*			-2.24	*0.003*	-1.81	*0.008*		
28	Superoxide dismutase [Fe], chloroplastic			2.09	*0.007*	1.96	*0.004*	2.67	*0.000*		
29	Glutathione S-transferase F9					2.02	*0.000*				
30	Vegetative storage protein 1					3.17	*0.000*				
31	Vegetative storage protein 1					2.76	*0.008*				
32	Ribulose bisphosphate carboxylase large chain			1.87	*0.036*					2.43	*0.006*
36	Ribulose bisphosphate carboxylase large chain	1.95	*0.008*	1.84	*0.031*	2.11	*0.025*			2.14	*0.030*
38	Ferredoxin--NADP reductase, leaf isozyme 2			2.02	*0.004*	2.3	*0.002*				
40	Aspartate-semialdehyde dehydrogenase									2.2	*0.004*
42	Ferredoxin--$NADP^+$ reductase	1.81	*0.022*	2.41	*0.005*	1.91	*0.029*	1.81	*0.063*	2.34	*0.013*
46	Malate dehydrogenase			3.14	*0.000*	3.08	*0.000*			2.23	*0.000*
47	Fructose-bisphosphate aldolase			5.5	*0.002*	4.48	*0.007*	3.33	*0.009*	3.17	*0.004*
48	Chloroplast stem-loop binding protein-41	2.14	*0.007*			2.73	*0.000*	2.08	*0.003*	1.92	*0.007*
49	Uroporphyrinogen decarboxylase 2			2.07	*0.032*	2.5	*0.007*				
50	ATP synthase gamma chain 1	1.95	*0.006*							2.23	*0.004*
51	Glyceraldehyde 3-phosphate dehydrogenase									1.82	*0.078*
52	RNA binding protein CSP41B	2.05	*0.018*			2.33	*0.016*			2.13	*0.004*
53	Glyceraldehyde-3-phosphate dehydrogenase	2.5	*0.011*	3.07	*0.000*	3.6	*0.001*			2.92	*0.001*
54	Glyceraldehyde-3-phosphate dehydrogenase			2.84	*0.003*	2.57	*0.000*			2.01	*0.001*

Supplementary Table S6 continued.

No[a]	Protein	250 Hz		500 Hz		1000 Hz		2000 Hz		3000 Hz	
		**Fold change**	***P* value**	**Fold change**	***P* value**	**Fold change**	***P* value**	**Fold change**	***P* value**	**Fold change**	***P* value**
	**1 h**										
57	Epithiospecifier modifier 1			2.25	*0.042*						
61	Phosphoribulokinase									2.69	*0.008*
62	Ribulose bisphosphate carboxylase/oxygenase activase	1.75	*0.012*	1.8	*0.008*	1.63	*0.015*	2.51	*0.002*	2.25	*0.002*
63	Phosphoglycerate kinase	1.85	*0.013*	2.58	*0.003*	2.45	*0.001*	1.91	*0.004*	2.46	*0.001*
64	Elongation factor Tu, chloroplastic	1.83	*0.009*			1.79	*0.001*				
65	S-adenosylmethionine synthase 4			1.74	*0.014*	1.89	*0.005*			2.16	*0.000*
66	Chaperonin 60 subunit beta 2									1.94	*0.001*
67	Myrosinase 1	2.88	*0.001*								
69	Glyceraldehyde-3-phosphate dehydrogenase B									3.83	*0.005*
71	Glyceraldehyde-3-phosphate dehydrogenase B			3.59	*0.001*					2.41	*0.004*
73	Monodehydroascorbate reductase	1.87	*0.017*	2.29	*0.003*	3.17	*0.001*	2.49	*0.004*	2.31	*0.003*
74	Glutamate-glyoxylate aminotransferase 1	2.2	*0.001*					1.78	*0.002*	2.32	*0.001*
	**24 h**										
1	RuBisCO small subunit 2B			-2.75	*0.000*	-1.93	*0.000*	-1.81	*0.008*	-2.22	*0.002*
5	F-type H^+-transporting ATPase subunit delta			2.64	*0.001*	1.73	*0.006*	2.43	*0.003*		
6	Peptidyl-prolyl cis-trans isomerase CYP20-3							1.92	*0.020*		
9	Nucleoside diphosphate kinase II									2.24	*0.007*
10	Nucleoside diphosphate kinase Ia									2.31	*0.009*
11	Putative major latex protein									1.8	*0.001*
12	Nucleoside diphosphate kinase 1			-1.92	*0.018*	-2.47	*0.003*				
13	Cytochrome b6-f complex iron-sulfur subunit	2.9	*0.001*	2.22	*0.024*	1.78	*0.017*			2.06	*0.021*
14	2-Cys peroxiredoxin BAS1									3.29	*0.002*
17	ATP synthase subunit beta	1.84	*0.006*					1.79	*0.002*	-2.28	*0.005*
19	Carbonic anhydrase 2, chloroplastic	1.91	*0.007*	2.18	*0.003*	2.06	*0.001*	2.2	*0.001*	2.15	*0.007*

Supplementary Table S6 continued.

No[a]	Protein	250 Hz		500 Hz		1000 Hz		2000 Hz		3000 Hz	
		Fold change	*P* value	Fold change	*P* value	Fold change	*P* value	Fold change	*P* value	Fold change	*P* value
	**24 h**										
20	AT2G37660					1.89	*0.004*	1.8	*0.001*	2.14	*0.000*
21	Carbonic anhydrase			3.88	*0.016*	4.5	*0.001*			3.5	*0.001*
24	Proteasome subunit beta type-6									2.33	*0.020*
25	L-ascorbate peroxidase			2.85	*0.009*	3.38	*0.006*			2.77	*0.001*
27	Glutathione S-transferase F2	2.36	*0.001*							1.91	*0.000*
30	Vegetative storage protein 1	2.15	*0.001*			1.9	*0.002*			2.3	*0.001*
31	Vegetative storage protein 1					2.42	*0.018*			2.71	*0.003*
32	Ribulose bisphosphate carboxylase large chain			1.83	*0.002*					2.07	*0.001*
33	Ribulose 1,5-bisphosphate carboxylase/oxygenase large chain			-1.87	*0.026*						
36	Ribulose bisphosphate carboxylase large chain	2.74	*0.007*								
40	Aspartate-semialdehyde dehydrogenase							1.9	*0.012*	2.2	*0.028*
42	Ferredoxin--NADP+ reductase					2.36	*0.004*	1.8	*0.066*		
45	Cysteine synthase					2.5	*0.008*			2.3	*0.010*
46	Malate dehydrogenase			2.23	*0.000*			1.9	*0.001*	1.9	*0.001*
47	Fructose-bisphosphate aldolase			3.33	*0.006*	2.56	*0.012*	2.44	*0.020*	2.22	*0.039*
48	Chloroplast stem-loop binding protein-41	2.67	*0.005*	2.29	*0.004*	2.61	*0.001*	1.97	*0.004*	2.51	*0.001*
49	Uroporphyrinogen decarboxylase 2									2.33	*0.013*
50	ATP synthase gamma chain 1			2.4	*0.012*			2.23	*0.005*	2.45	*0.003*
51	Glyceraldehyde 3-phosphate dehydrogenase	2.29	*0.003*	1.86	*0.035*	1.79	*0.010*	2.32	*0.010*	1.86	*0.018*
52	RNA binding protein CSP41B	2.01	*0.017*			1.81	*0.044*	2.18	*0.011*	2.12	*0.012*
57	Epithiospecifier modifier 1							2.17	*0.021*		
58	Glutamine synthetase cytosolic isozyme 1-1					1.82	*0.079*	2.35	*0.023*	1.94	*0.090*
59	Glutamine synthetase			4.05	*0.002*						
60	Glutamine synthetase			4.79	*0.002*						

Supplementary Table S6 continued.

No[a]	Protein	250 Hz		500 Hz		1000 Hz		2000 Hz		3000 Hz	
		**Fold change**	***P* value**	**Fold change**	***P* value**	**Fold change**	***P* value**	**Fold change**	***P* value**	**Fold change**	***P* value**
	**24 h**										
61	Phosphoribulokinase			2.27	0.014	1.82	0.008	1.91	0.010	2.64	0.010
62	Ribulose bisphosphate carboxylase /oxygenase activase			1.93	0.003	1.74	0.006	2.13	0.008		
63	Phosphoglycerate kinase			2.78	0.000	2.6	0.001	3.38	0.001	3.79	0.003
64	Elongation factor Tu, chloroplastic			3.87	0.000	2.24	0.002	2.59	0.002	2.28	0.010
66	Chaperonin 60 subunit beta 2					2.92	0.000	2.53	0.002	1.97	0.002
67	Myrosinase 1							-1.96	0.009	-2.56	0.002
69	Glyceraldehyde-3-phosphate dehydrogenase B							2.44	0.016	2.33	0.004
70	ATP sulfurylase 1			1.91	0.009	1.88	0.018	2.39	0.012	2.48	0.007
71	Glyceraldehyde-3-phosphate dehydrogenase B					2.36	0.000	1.98	0.001	2.02	0.006
72	Isocitrate dehydrogenase			2.38	0.002	1.83	0.005			2.17	0.042
73	Monodehydroascorbate reductase	1.85	0.007	1.92	0.025	2.22	0.003	2.38	0.008	2.45	0.008
74	Glutamate-glyoxylate aminotransferase 1	2.22	0.016	2.15	0.032	1.71	0.023	2.03	0.005	3.36	0.002
75	NADP-dependent glyceraldehyde-3-phosphate dehydrogenase					3.56	0.044			2.92	0.018
76	Serine hydroxymethyltransferase					3.75	0.000	2.83	0.050	3.5	0.001
78	Serine hydroxymethyltransferase			4.6	0.034	5.7	0.002	3.8	0.036	4.6	0.000
	**48 h**										
1	RuBisCO small subunit 2B			-2.71	0.001	-3.19	0.000	-2.42	0.001	-2.58	0.000
4	RuBisCO small subunit 1A, chloroplastic			-2.86	0.001	-3.23	0.001	-2.66	0.001	-2.66	0.001
5	F-type H+-transporting ATPase subunit delta	1.92	0.003			2.11	0.005	2.08	0.044	1.97	0.021
8	Eukaryotic translation initiation factor 5A-2					2.45	0.036				
12	Nucleoside diphosphate kinase 1					-1.77	0.045	-1.84	0.039	-2.19	0.026
13	Cytochrome b6-f complex iron-sulfur subunit			-1.94	0.042	-2.33	0.028	-1.94	0.035	-2.26	0.031
15	Ribose 5-phosphate isomerase A			2.07	0.008						

Supplementary Table S6 continued.

No[a]	Protein	250 Hz		500 Hz		1000 Hz		2000 Hz		3000 Hz	
		Fold change	*P* value	Fold change	*P* value	Fold change	*P* value	Fold change	*P* value	Fold change	*P* value
						**48 h**					
17	ATP synthase subunit beta	2.19	*0.005*								
18	Oxygen-evolving enhancer protein 1-2			2.39	*0.001*	2.5	*0.026*				
19	Carbonic anhydrase 2, chloroplastic					2.53	*0.009*			-1.89	*0.026*
20	AT2G37660					2.29	*0.002*			-1.8	*0.011*
21	Carbonic anhydrase					2.3	*0.005*	2.5	*0.078*		
22	Myb domain protein 17									-2.34	*0.008*
23	Glutathione S-transferase DHAR2			2.38	*0.000*						
24	Proteasome subunit beta type-6			1.89	*0.082*	2.89	*0.003*				
25	L-ascorbate peroxidase			3.23	*0.001*	3.38	*0.001*	2.15	*0.014*		
26	Germin-like protein subfamily 3			-2.46	*0.001*	-1.72	*0.005*	-2.08	*0.003*	-2.16	*0.005*
27	Glutathione S-transferase F2	-1.9	*0.006*							-1.94	*0.005*
28	Superoxide dismutase [Fe], chloroplastic	-1.83	*0.011*							-1.98	*0.005*
30	Vegetative storage protein 1	-2.03	*0.018*	2.1	*0.013*			-2.03	*0.015*	-1.97	*0.012*
31	Vegetative storage protein 1	-2.21	*0.030*	2.27	*0.017*			-2.37	*0.013*	-1.83	*0.038*
32	Ribulose bisphosphate carboxylase large chain			1.93	*0.035*	2.79	*0.020*			1.93	*0.020*
34	AT5G02240	-1.83	*0.025*			2.51	*0.002*				
35	V-type proton ATPase subunit E1					2.48	*0.023*				
37	Ferredoxin--NADP reductase, leaf isozyme 2					2.41	*0.003*				
38	Ferredoxin--NADP reductase, leaf isozyme 2			3.83	*0.001*					-2.08	*0.035*
40	Aspartate-semialdehyde dehydrogenase					1.9	*0.002*	2.3	*0.022*	2.9	*0.048*
43	Phosphoglycerate kinase			2.67	*0.016*	2.37	*0.036*	2	*0.042*	2.22	*0.026*
44	Phosphoglycerate kinase						*0.174*			1.79	*0.009*
45	Cysteine synthase	2.65	*0.000*	1.93	*0.007*	2.22	*0.006*	1.85	*0.022*	2.29	*0.002*
46	Malate dehydrogenase			2.4	*0.000*	3.26	*0.001*	2.25	*0.002*	2.06	*0.002*
47	Fructose-bisphosphate aldolase			2.36	*0.025*	1.91	*0.004*	3.45	*0.010*	2.45	*0.020*
48	Chloroplast stem-loop binding protein-41			2.66	*0.001*	2.76	*0.000*	2.38	*0.001*	2.23	*0.002*

Supplementary Table S6 continued.

No[a]	Protein	250 Hz		500 Hz		1000 Hz		2000 Hz		3000 Hz	
		**Fold change**	***P* value**	**Fold change**	***P* value**	**Fold change**	***P* value**	**Fold change**	***P* value**	**Fold change**	***P* value**
	**48 h**										
49	Uroporphyrinogen decarboxylase 2			2.17	*0.033*	2.5	*0.003*				
50	ATP synthase gamma chain 1			2.55	*0.019*	2.18	*0.032*				
51	Glyceraldehyde 3-phosphate dehydrogenase			2.25	*0.009*	2.38	*0.029*				
52	RNA binding protein CSP41B	2.33	*0.005*	2.24	*0.009*	2.64	*0.010*	2.39	*0.030*	2.3	*0.008*
53	Glyceraldehyde-3-phosphate dehydrogenase			2.33	*0.013*	1.92	*0.007*	2.11	*0.009*	2.06	*0.003*
54	Glyceraldehyde-3-phosphate dehydrogenase			2.12	*0.005*	2.23	*0.010*	1.85	*0.007*	2.14	*0.003*
55	Hydroxypyruvate reductase	1.91	*0.058*								
56	Glyceraldehyde-3-phosphate dehydrogenase			2.67	*0.037*	2.33	*0.013*	2.22	*0.007*		
57	Epithiospecifier modifier 1			1.88	*0.003*	2.67	*0.000*				
61	Phosphoribulokinase					1.78	*0.012*	2.11	*0.004*	2.67	*0.015*
62	Ribulose bisphosphate carboxylase /oxygenase activase					2.4	*0.005*				
63	Phosphoglycerate kinase	1.79	*0.010*	2.03	*0.032*	1.83	*0.028*			1.76	*0.034*
65	S-adenosylmethionine synthase 4						*0.212*			1.95	*0.012*
66	Chaperonin 60 subunit beta 2					2.17	*0.005*	1.97	*0.005*		
68	Phosphoglycerate dehydrogenase-like protein					2.08	*0.029*	2.92	*0.010*		
69	Glyceraldehyde-3-phosphate dehydrogenase B							1.9	*0.017*	2.1	*0.019*
71	Glyceraldehyde-3-phosphate dehydrogenase B			2.37	*0.011*	2.13	*0.034*	1.87	*0.007*	2.32	*0.035*
72	Isocitrate dehydrogenase			2.44	*0.021*	2.63	*0.025*	2	*0.040*		
73	Monodehydroascorbate reductase			1.85	*0.032*	1.89	*0.029*	1.7	*0.027*		
74	Glutamate-glyoxylate aminotransferase 1			2.2	*0.008*	2.71	*0.003*	2.8	*0.005*	2.43	*0.016*
75	NADP-dependent glyceraldehyde-3-phosphate dehydrogenase							1.95	*0.041*	2.4	*0.029*
76	Serine hydroxymethyltransferase							1.92	*0.007*	2.77	*0.002*
78	Serine hydroxymethyltransferase							3.47	*0.002*	2.93	*0.034*

Supplementary Table S7. Showing functional annotations of differentially expressed proteins based on Mapman annotation of the plant proteome database (PPDB). [a] indicates the numbers correspond to the 2-DE gels shown in Supplementary Fig. S3.

No[a]	Protein	Mapman annotation
1	RuBisCO small subunit 2B	1.3.2 PS.calvin cyle.rubisco small subunit
2	RuBisCO small subunit 1A	1.3.2 PS.calvin cyle.rubisco small subunit
3	40S ribosomal protein S20-1	29.2.1.2.1.20 protein.synthesis.ribosomal protein.eukaryotic.40S subunit.S20
4	RuBisCO small subunit 1A, chloroplastic	1.3.2 PS.calvin cyle.rubisco small subunit
5	F-type H+-transporting ATPase subunit delta	1.1.4 PS.lightreaction.ATP synthase
6	Peptidyl-prolyl cis-trans isomerase CYP20-3	29.6 protein.folding
7	Peptidyl-prolyl cis-trans isomerase CYP20-3	29.6 protein.folding
8	Eukaryotic translation initiation factor 5A-2	29.2.3 protein.synthesis.initiation
9	Nucleoside diphosphate kinase II	23.4.10 nucleotide metabolism.phosphotransfer and pyrophosphatases. nucleoside diphosphate kinase
10	Nucleoside diphosphate kinase Ia	23.4.10 nucleotide metabolism.phosphotransfer and pyrophosphatases. nucleoside diphosphate kinase
11	Putative major latex protein	20.2.99 stress.abiotic.unspecified
12	Nucleoside diphosphate kinase 1	23.4.10 nucleotide metabolism.phosphotransfer and pyrophosphatases. nucleoside diphosphate kinase
13	Cytochrome b6-f complex iron-sulfur subunit	1.1.3 PS.lightreaction.cytochrome b6/f
14	2-Cys peroxiredoxin BAS1	21.5 redox.periredoxins
15	Ribose 5-phosphate isomerase A	1.3.10 PS.calvin cyle.Rib5P Isomerase
16	Light harvesting chlorophyll a/b binding protein	1.1.1.1 PS.lightreaction.photosystem II.LHC-II
17	ATP synthase subunit beta	1.1.4 PS.lightreaction.ATP synthase
18	Oxygen-evolving enhancer protein 1-2	1.1.1.2 PS.lightreaction.photosystem II
19	Carbonic anhydrase 2, chloroplastic	8.3 TCA / org.transformation.carbonic anhydrases
20	AT2G37660	35.2 not assigned.unknown
21	Carbonic anhydrase	8.3 TCA / org.transformation.carbonic anhydrases
22	Myb domain protein 17	27.3.25 RNA.regulation of transcription.MYB domain transcription factor family
23	Glutathione S-transferase DHAR2	21.2.1 redox.ascorbate and glutathione.ascorbate
24	Proteasome subunit beta type-6	29.5.11.20 protein.degradation.ubiquitin.proteasome.20S
25	L-ascorbate peroxidase	21.2.1 redox.ascorbate and glutathione.ascorbate

Supplementary Table S7 continued.

No[a]	Protein	Mapman annotation
26	Germin-like protein subfamily 3	20.2.99 stress.abiotic.unspecified
27	Glutathione S-transferase F2	26.9 misc.glutathione S transferases
28	Superoxide dismutase [Fe], chloroplastic	21.6 redox.dismutases and catalases
29	Glutathione S-transferase F9	26.9 misc.glutathione S transferases
30	Vegetative storage protein 1	33.1 development.storage proteins
31	Vegetative storage protein 1	33.1 development.storage proteins
32	Ribulose bisphosphate carboxylase large chain	1.3.1 PS.calvin cyle.rubisco large subunit
33	Ribulose 1,5-bisphosphate carboxylase /oxygenase large chain	1.3.1 PS.calvin cyle.rubisco large subunit
34	AT5G02240	35.2 not assigned.unknown
35	V-type proton ATPase subunit E1	34.1.1 transport.p- and v-ATPases.H^+-transporting two-sector ATPase
36	Ribulose bisphosphate carboxylase large chain	1.3.1 PS.calvin cyle.rubisco large subunit
37	Ferredoxin--NADP reductase, leaf isozyme 2	1.1.7 PS.lightreaction.ferredoxin reductase
38	Ferredoxin--NADP reductase, leaf isozyme 2	1.1.7 PS.lightreaction.ferredoxin reductase
39	Fructose-bisphosphate aldolase	1.3.6 PS.calvin cyle.aldolase
40	Aspartate-semialdehyde dehydrogenase	13.1.3.6.1.2 amino acid metabolism.synthesis.aspartate family.misc.homoserine.aspartate semialdehyde dehydrogenase
41	Probable fructose-bisphosphate aldolase 2	1.3.6 PS.calvin cyle.aldolase
42	Ferredoxin--$NADP^+$ reductase	1.1.7 PS.lightreaction.ferredoxin reductase
43	Phosphoglycerate kinase	4.10 glycolysis.phosphoglycerate kinase
44	Phosphoglycerate kinase	4.10 glycolysis.phosphoglycerate kinase
45	Cysteine synthase	13.1.5.3.1 amino acid metabolism.synthesis.serine-glycine-cysteine group. cysteine.OASTL
46	Malate dehydrogenase	8.2.9 TCA / org.transformation.other organic acid transformaitons.cyt MDH
47	Fructose-bisphosphate aldolase	4.7 glycolysis.aldolase
48	Chloroplast stem-loop binding protein-41	27.3.99 RNA.regulation of transcription.unclassified
49	Uroporphyrinogen decarboxylase 2	19.7 tetrapyrrole synthesis.uroporphyrinogen decarboxylase
50	ATP synthase gamma chain 1	1.1.4 PS.lightreaction.ATP synthase
51	Glyceraldehyde 3-phosphate dehydrogenase	4.9 glycolysis.glyceraldehyde 3-phosphate dehydrogenase

Supplementary Table S7 continued.

No[a]	Protein	Mapman annotation
52	RNA binding protein CSP41B	27 RNA
53	Glyceraldehyde-3-phosphate dehydrogenase	4.9 glycolysis.glyceraldehyde 3-phosphate dehydrogenase
54	Glyceraldehyde-3-phosphate dehydrogenase	4.9 glycolysis.glyceraldehyde 3-phosphate dehydrogenase
55	Hydroxypyruvate reductase	1.2.6 PS.photorespiration.hydroxypyruvate reductase
56	Glyceraldehyde-3-phosphate dehydrogenase A	1.3.4 PS.calvin cyle.GAP
57	Epithiospecifier modifier 1	26.28 misc.GDSL-motif lipase
58	Glutamine synthetase cytosolic isozyme 1-1	12.2.2 N-metabolism.ammonia metabolism.glutamine synthase
59	Glutamine synthetase	12.2.2 N-metabolism.ammonia metabolism.glutamine synthase
60	Glutamine synthetase	12.2.2 N-metabolism.ammonia metabolism.glutamine synthase
61	Phosphoribulokinase	1.3.12 PS.calvin cyle.PRK
62	Ribulose bisphosphate carboxylase /oxygenase activase	1.3.13 PS.calvin cyle.rubisco interacting
63	Phosphoglycerate kinase	4.10 glycolysis.phosphoglycerate kinase
64	Elongation factor Tu, chloroplastic	29.2.4 protein.synthesis.elongation
65	S-adenosylmethionine synthase 4	13.1.3.4.11 amino acid metabolism.synthesis.aspartate family.methionine.S-adenosylmethionine synthetase
66	Chaperonin 60 subunit beta 2	29.6 protein.folding
67	Myrosinase 1	16.5.1 secondary metabolism.sulfur-containing.glucosinolates
68	Phosphoglycerate dehydrogenase-like protein	13.1.5.1.1 amino acid metabolism.synthesis.serine-glycine-cysteine group.serine. phosphoglycerate dehydrogenase
69	Glyceraldehyde-3-phosphate dehydrogenase B	1.3.4 PS.calvin cyle.GAP
70	ATP sulfurylase 1	14.1 S-assimilation.APS
71	Glyceraldehyde-3-phosphate dehydrogenase B	1.3.4 PS.calvin cyle.GAP
72	Isocitrate dehydrogenase	8.1.4 TCA / org.transformation.TCA.IDH
73	Monodehydroascorbate reductase	21.2.1 redox.ascorbate and glutathione.ascorbate
74	Glutamate-glyoxylate aminotransferase 1	1.2.3 PS.aminotransferases peroxisomal
75	NADP-dependent glyceraldehyde-3-phosphate dehydrogenase	2 major CHO metabolism
76	Serine hydroxymethyltransferase	25.1 C1-metabolism.glycine hydroxymethyltransferase

Supplementary Table S7 continued.

No[a]	Protein	Mapman annotation
77	Catalase	21.6 redox.dismutases and catalases
78	Serine hydroxymethyltransferase	25.1 C1-metabolism.glycine hydroxymethyltransferase

Supplementary Table S8. List of Real time PCR primers.

Gene ID	Forward Primer	Reverse Primer
At1g76650 (CML38)	AGATGACGAAGAAGAGAAGAAGATG	CTTAGCGCATCATAAGAGCAAACT
At3g50060 (MYB77)	GATGAAGATTAATGTAGAGGAGAGA GG	TCAACCTTAGGTGTTATTACTCCAC
At1g76600	GACGTTGAATCAGGCTAACGATAAT	GAGAGTCTCAGTCTAAACGATCGAA
At1g25400	GGCATGGTAGGGTCTAATAAAGACT	ATACACGACAACAGGAGGTTGAAT
At1g33720 (CYP76C6)	AGGTACAGATTATGAGCTTACACCG	ATTTATGGCTCGTTTCTTCAGAGG
At1g01560 (MPK11)	CTAGATTCCCAAACATGTCGGTC	TATAAGCTCCTTGATGTTCTCTTCC
At3g07350	AAATGTTGTCGTCTCTCGGATCT	GACGAGAAATCTAAATCGACGATGA
At3g27690 (LHCB2)	GATTCTTTGTCCAAGCCATAGTTAC	GGAACCGATGTATCAAATATAGGG
At2g20670	ACTGTAGCGGTCGTGTGATATTT	CTAATTTCTTCCAGCACCAATGAC
At1g21910 (DREB26)	CTCAAGCTGCCAACTCATTTAAC	CGTCGAGTTTAGTTGAGGAGAGTAA
At2g44500	CTTACGCTGGTCACAAGAAATACAT	CTGTTGTTCTTGTTGTTGTCTATCC
At2g40000 (HSPRO2)	GGAGGACCAAGATTACAAGAGGC	GGCTCCATAAATATCTGACTCAGTG
At1g13260 (RAV1)	GGTGACGTGGTTAGTTTCAGTAGAT	CTACACACCAACGATAACATCTCAG
At1g70290 (TPS8)	GAGACGATAGATCAGACGAAGACAT	CAACTTGTGTGTGAGAAGAACTAGG
At4g37610 (BT5)	CGTGTAATGGTTTAGAGCAGCTATT	CTCATCTTTCCTGCTCTGTGTCT
At1g80440 (KMD1)	GTGGACACAACGAAGAGAAATGT	ACTCCCACGTAGACACATCGAAC
At5g22920 (RZPF34)	GTGACATGTCTAATCTGTGGAAGAA	ACTATCAGAGCCTCTCTGTGTCTGT
At1g13440 (GAPDH)	GAGAGTTTGTGTGTGGTTGAGTTC	GGTTTGAGTTAGCACGAGAAAGTAA

Supplementary Figure S1. Schematic representation of sound vibration (SV) treatment and sample harvest strategy. Five different frequencies (250, 500, 1000, 2000 and 3000 Hertz) were separately applied with constant amplitude (80 decibels) to Arabidopsis for 1 h. qRT-PCR, quantitative real-time PCR; 2-DGE, 2-dimensional gel electrophoresis.

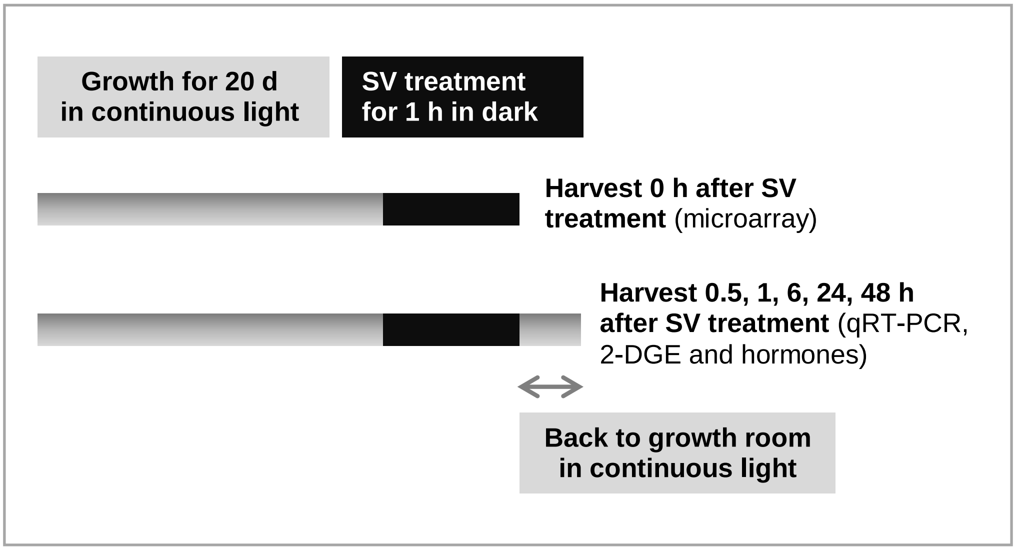

Supplementary Figure S2. Gene Ontology (GO) analysis of differentially expressed genes (fold change > 2, $P < 0.05$) identified by microarray. (a) Cellular component. (b) Molecular function. (c) Biological process. U and D represent up-regulated and down-regulated genes, respectively. Five different frequencies (250, 500, 1000, 2000, 3000 Hertz) are marked numerically (1, 2, 3, 4 and 5, respectively). Classification was made by TAIR Gene Ontology annotation tool (http://www.arabidopsis.org).

(a)

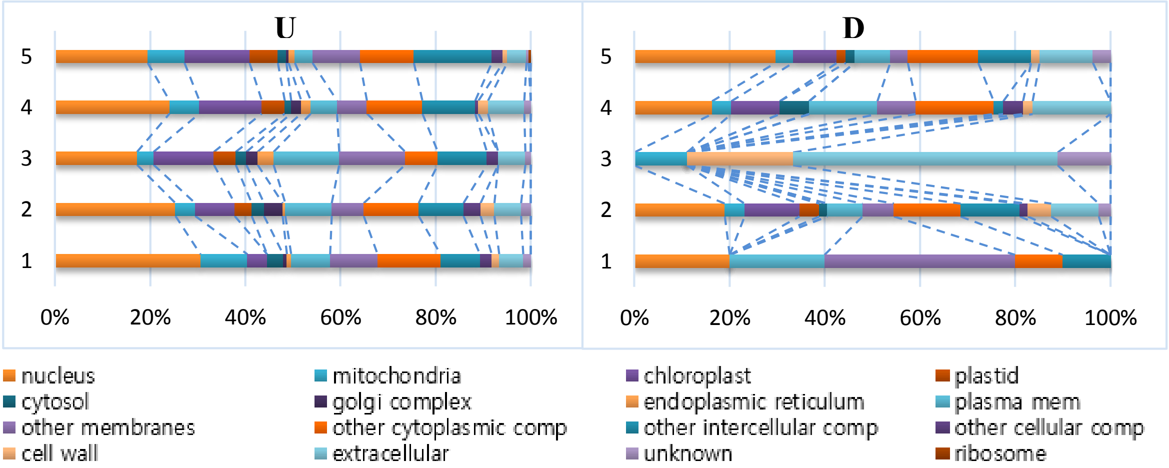

(b)

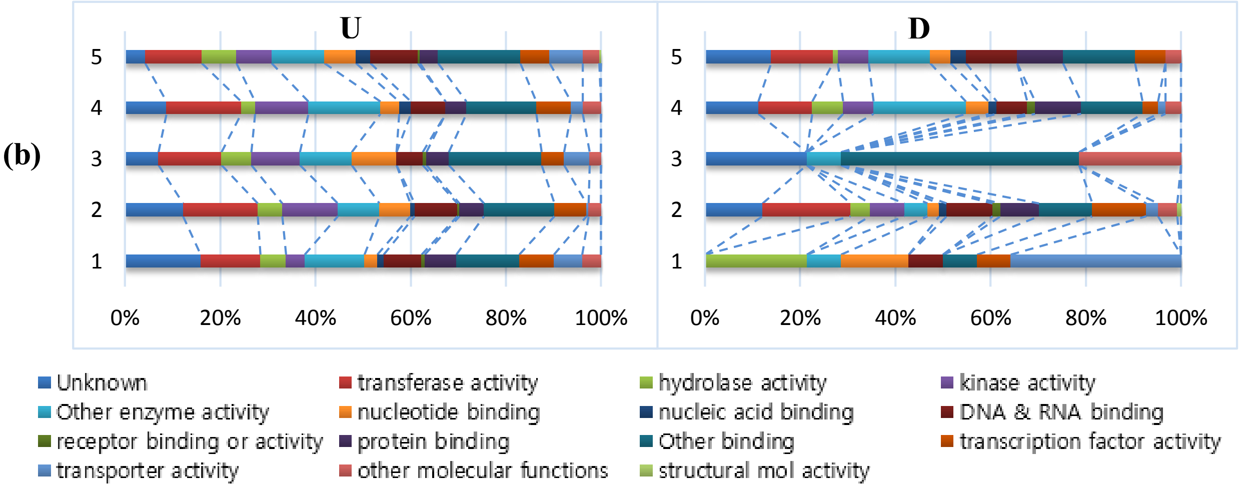

(c)

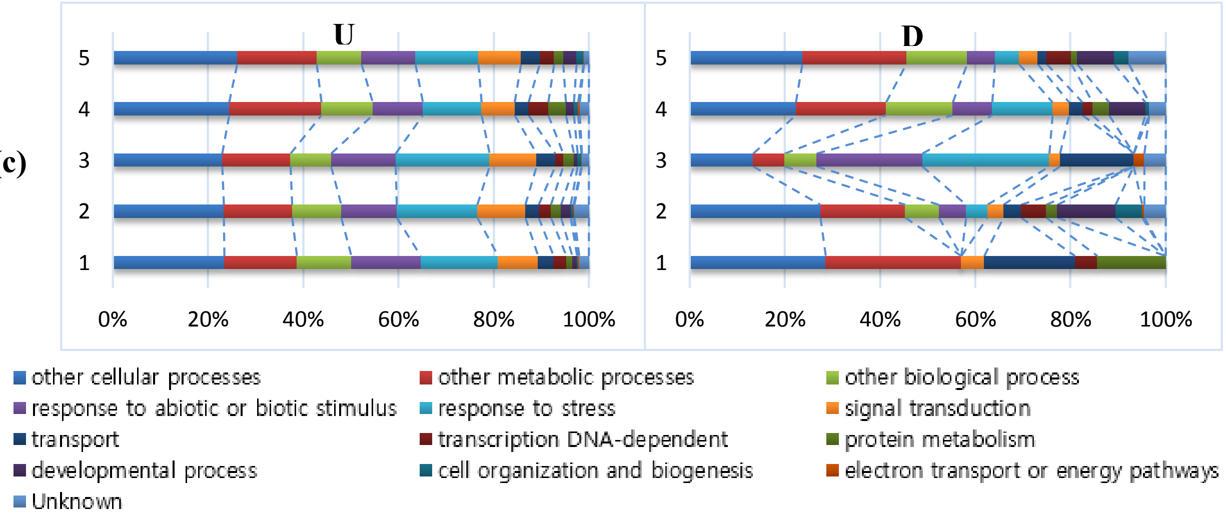

Supplementary Figure S3. Representative 2D gel electrophoresis image for proteomic analysis. Five different frequencies (250, 500, 1000, 2000 and 3000 Hertz) were separately applied to Arabidopsis for 1 h with 80 decibels and samples were harvested at 0, 1, 24 and 48 h.

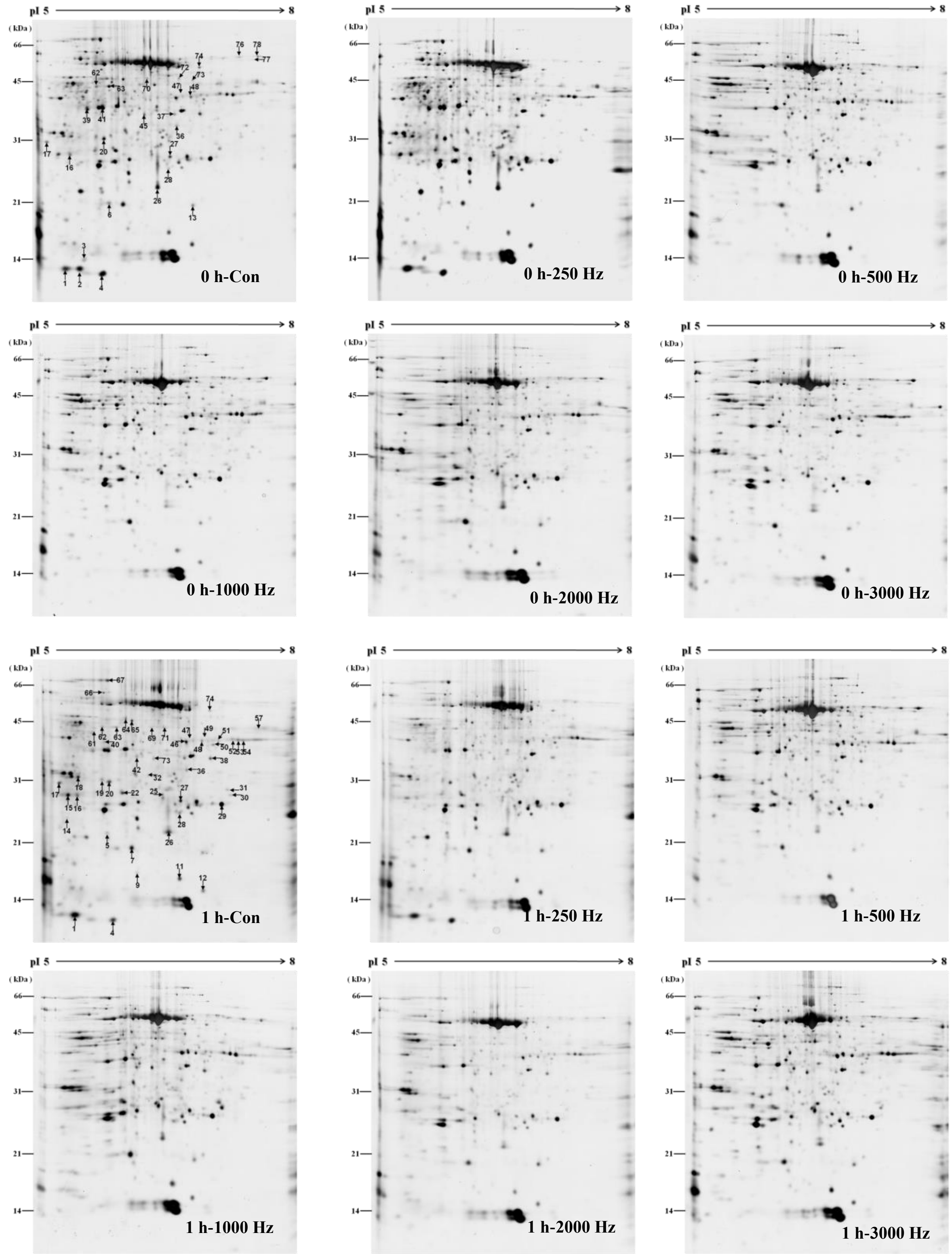

Supplementary Figure S3 continued.

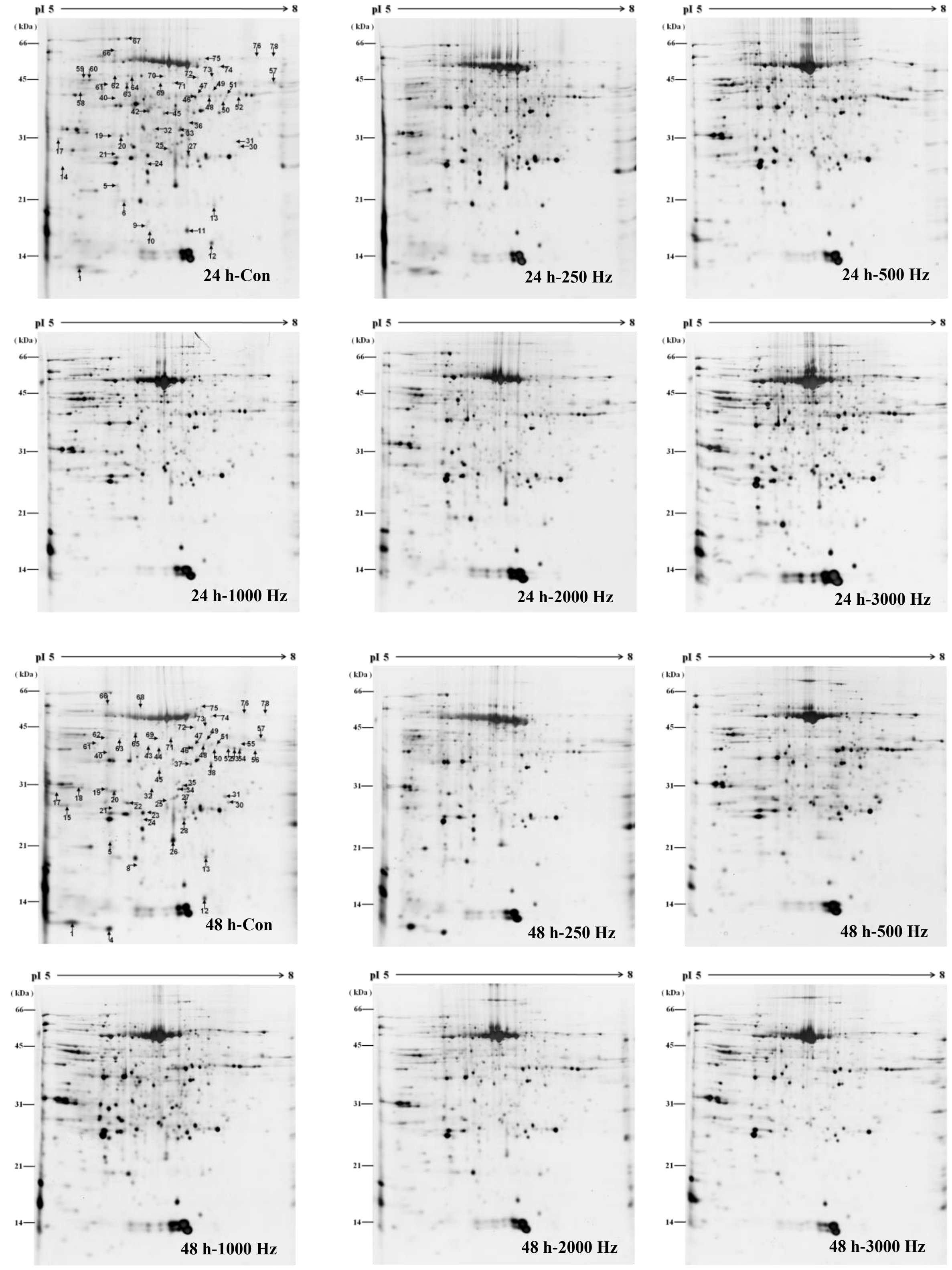

Supplementary Figure S4. Gene Ontology (GO) analysis of differentially expressed proteins identified by MALDI-TOF/MS. (a) Biological process. (b) Cellular component. (c) Molecular function. Five different frequencies (250, 500, 1000, 2000 and 3000 Hertz) were separately applied to Arabidopsis for 1 h with 80 decibels.

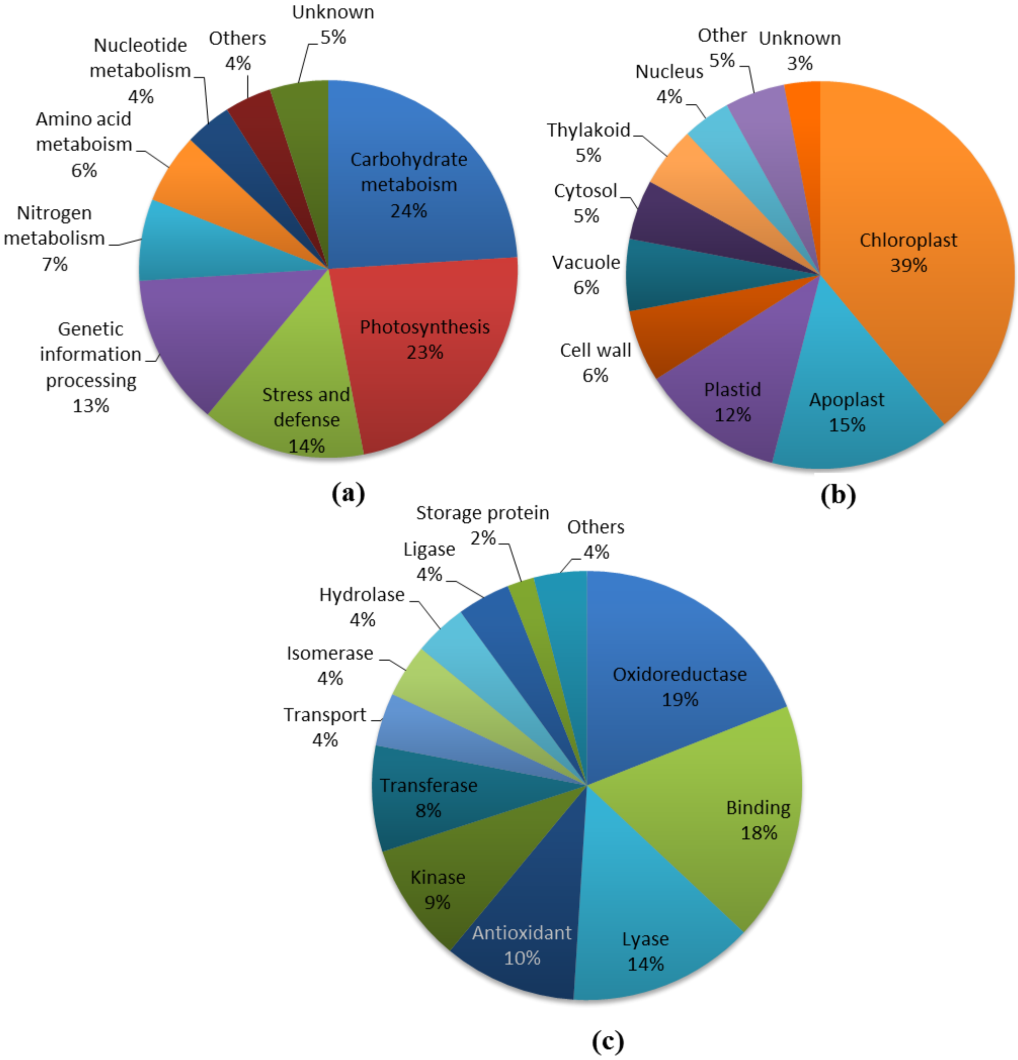

Supplementary Figure S5. Hormone measurement by LC-MS after the 500 Hertz (80 decibels) treatment. Error bar indicates the standard deviation of mean obtained from two replicates. Each replicate represented more than one plant. *P*-value ranges are marked by asterisks: ** $P < 0.05$, * $P < 0.1$.

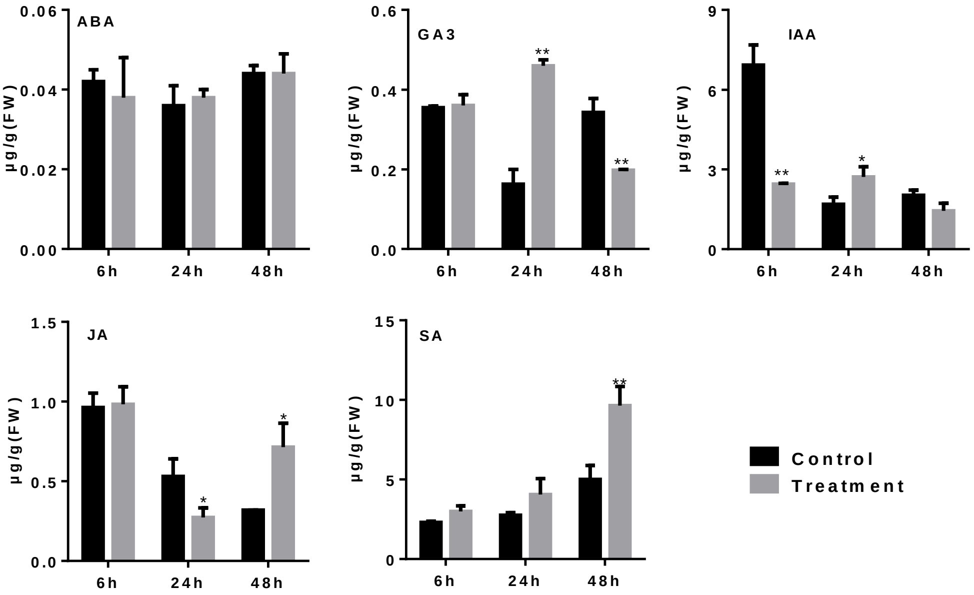

Supplement: Supplementary Information [file srep33370-s1.pdf]
